# Crystallographic Origins of Temperature-Dependent Strain Rate Sensitivity and Deformation Mechanism Transitions at TATB/F2314 Interfaces: A Molecular Dynamics Study

**DOI:** 10.3390/polym18212692

**Published:** 2026-10-30

**Authors:** Wendi Shi, Tingting Zhou, Yao Long, Jun Chen, Anmin He, Pei Wang

**Affiliations:** 1 National Key Laboratory of Computational Physics, Institute of Applied Physics and Computational Mathematics, Beijing 100094, China; shiwendi@mail.nwpu.edu.cn (W.S.); jun_chen@iapcm.ac.cn (J.C.); he_anmin@iapcm.ac.cn (A.H.); wangpei@iapcm.ac.cn (P.W.); 2 Center for Applied Physics and Technology, Peking University, Beijing 100871, China

**Keywords:** interface, crystallographic orientation, deformation mechanism, PBX, molecular dynamics

## Abstract

Interfaces play a critical role in the mechanical integrity of polymer-bonded explosives (PBXs) based on 1,3,5-triamino-2,4,6-trinitrobenzene (TATB), yet how crystallographic orientation governs their temperature- and strain-rate-dependent mechanical response remains poorly understood. Here, molecular dynamics simulations were performed on interfaces between TATB and the fluoropolymer F2314, with TATB exposing the (001), (010), and (100) facets, over wide ranges of temperature and strain rate. Crystallographic orientation was found to regulate the mechanical response by determining deformation mechanisms. The edge-exposed (010) and (100) facets allowed basal-plane slip to develop within the available loading time, producing weakly temperature-dependent apparent strain rate sensitivities. In contrast, the planar (001) facet constrained basal deformation at low temperatures but underwent coupled structural rearrangement at 400 K, enabling kink band formation and increasing its rate sensitivity. Rapid loading kinetically suppressed these coordinated mechanisms and shifted deformation toward distributed molecular deformation. F2314 simultaneously accommodated heterogeneous deformation through rate- and temperature-dependent chain motion, coupling polymer relaxation to the orientation-dependent response of TATB. The competition between these processes further governed damage modes. As such, these findings establish a relationship between crystallographic geometry and interfacial deformation kinetics, providing an atomistic basis for multiscale modeling and microstructural design of TATB-based PBXs.

## 1. Introduction

Polymer-bonded explosives (PBXs), composed of more than 90 vol% high-energy particles embedded in less than 10 vol% polymeric binder, are widely used in both civil and military applications, including solid propellants and warhead charge payloads, owing to their high energy density, reliable safety performance, and adequate mechanical properties [1,2]. In addition to the routine mechanical loads imposed during manufacture, transport, and storage, the rising performance requirements of modern systems increasingly demand that PBXs bear structural loads and accommodate shape deformation as integral structural components [3]. Under such service conditions, PBXs are inevitably subjected to dynamic loading over extremely short durations. Their mechanical response is complex and strongly dependent on the microstructure, so a clear relationship between microstructure and property is essential for the structural design and safe use of PBXs. Among the governing microstructural features, the interface between the energetic particles and the binder is often the weakest region and plays a critical role in both the mechanical strength and the ignition safety of the material.

Despite its small volume fraction, the interface plays a decisive role in the mechanical behavior of PBXs. Under mechanical loading, interfacial failure frequently occurs, and macroscopic experiments have shown that cracks in PBXs propagate predominantly along interfaces [4]. In addition to degrading the macroscopic mechanical response, such debonding may generate localized hotspots that ignite the explosive and trigger detonation [5]. In other words, the interface governs both the load-bearing capacity and the ignition safety of PBXs. To describe this behavior, a number of continuum and mesoscale models have been developed. Cohesive zone models are based on the relationship between interfacial traction and separation and have been successfully used to simulate void nucleation in interfacial regions [6]. However, these models rely on phenomenological traction separation laws whose parameters are empirically calibrated rather than derived from the underlying physical mechanisms. Systematic atomistic studies are therefore required to elucidate the molecular mechanisms governing interfacial deformation and failure.

TATB is among the most widely used insensitive energetic crystals in PBXs. It inherently possesses a triclinic cell of space group $P\bar{1}$ [7], and its layered, graphite-like molecular structure makes its thermal, chemical, and mechanical properties strongly anisotropic. Numerous experimental and theoretical studies have been performed to characterize the origin of this anisotropy. Kolb and Rizzo [8] measured markedly different linear thermal expansion coefficients along the three principal crystallographic axes (*a*, *b* and *c*). Bedrov et al. [9] also reported considerable mechanical anisotropy by calculating the isothermal second-order elastic tensor and the corresponding bulk and shear moduli using molecular dynamics simulations. Chaudhuri et al. [10] further reported that interfacial adhesion energy depends on crystallographic orientation. In addition to altering interfacial adhesion, crystallographic orientation changes the geometric relationship between the loading direction and the layered molecular structure of TATB. Different crystal facets may therefore select distinct modes of molecular accommodation.

Crystallographic orientation, however, is not the only factor governing the interfacial response. In service, the interface is subjected to wide ranges of strain rates and temperatures, both of which strongly affect its structure and mechanical properties. Increasing temperature enhances polymer chain mobility and may cause the interface to transition from brittle to more ductile deformation [11]. In addition, interface structure usually exhibits obvious strain rate hardening ability, with the peak stress increasing markedly with strain rate. Previous molecular dynamics simulations of the TATB/F2314 interface established the existence of an intermixing region and demonstrated that temperature and strain rate affect its mechanical response [12]. However, whether these effects depend on the crystallographic facet exposed to F2314 remains unresolved. In particular, it remains unclear how TATB orientation governs the activation of deformation mechanisms. This issue is important because PBXs contain crystals with diverse orientations, and even a single irregular crystal may expose several facets to the surrounding binder.

To this end, molecular dynamics simulations were performed on TATB/F2314 interfaces, exposing the (001), (010), and (100) facets over wide ranges of temperature and strain rate. The relaxed structures, tensile responses, deformation behavior, and damage morphologies were systematically analyzed. We demonstrate that facet-dependent interfacial packing and crystallographic geometry govern the deformation mechanisms, whereas temperature and strain rate regulate their development. These findings advance the understanding of orientation-dependent deformation in TATB/polymer interfaces under thermomechanical loading.

## 2. Materials and Methods

**Figure 1:**
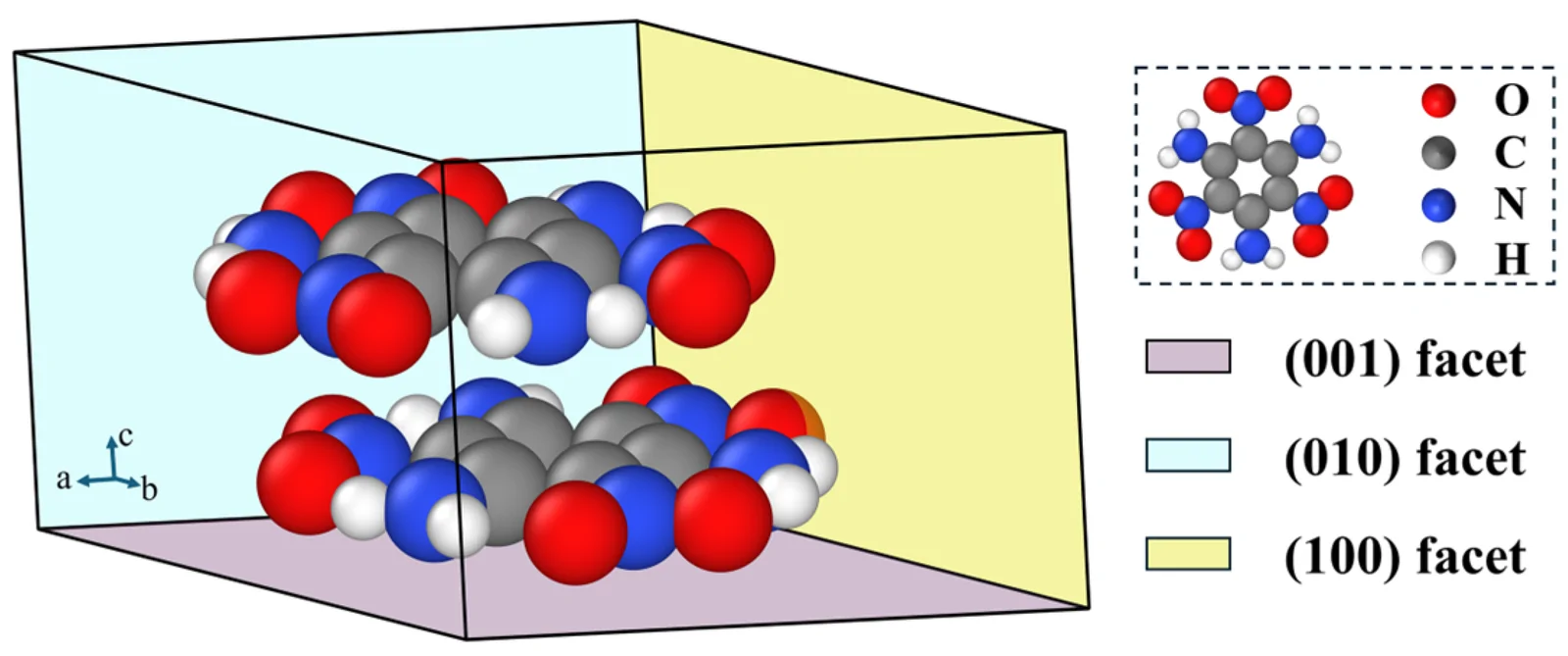
Schematic illustration of the layered molecular arrangement and crystallographic orientations of TATB. Two TATB molecules are shown in a triclinic unit cell with a staggered stacking configuration.

**Figure 2:**
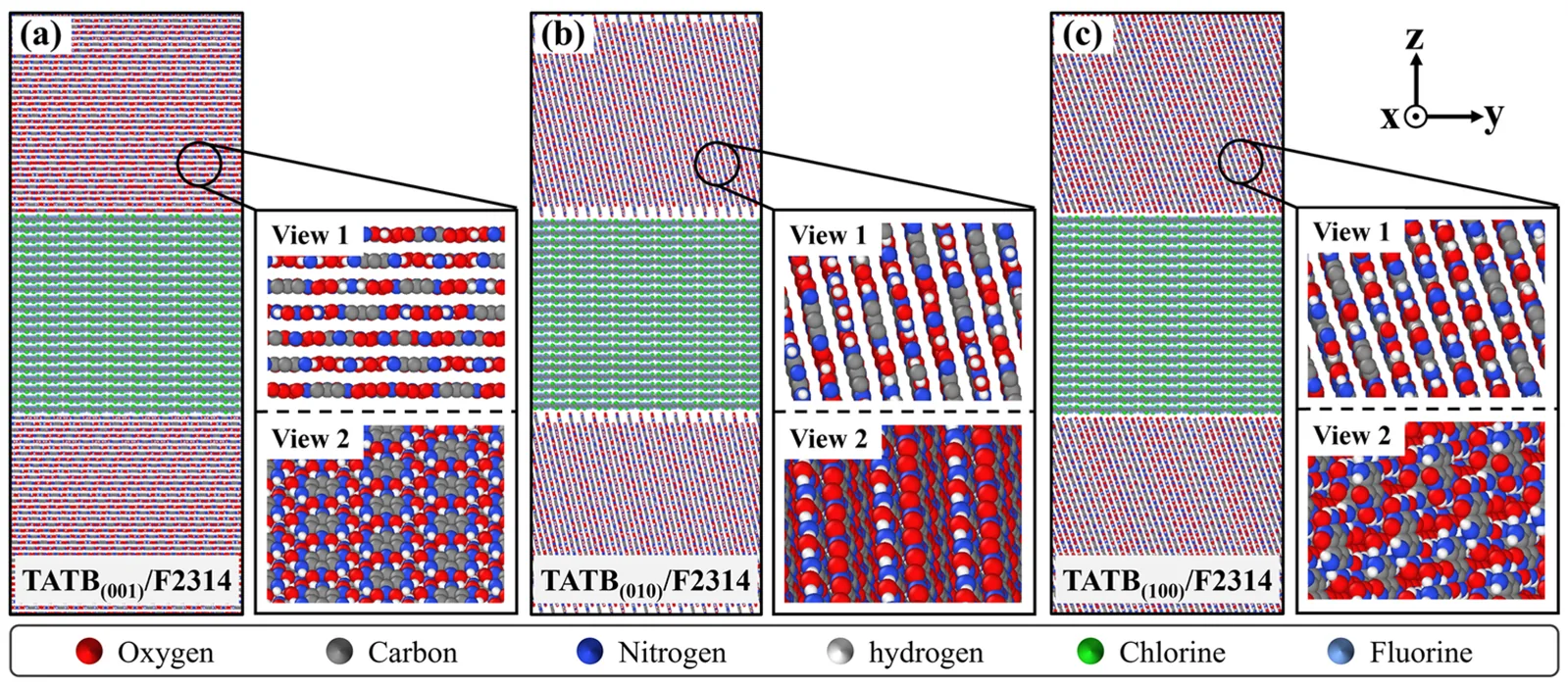
Interface models with different crystallographic orientations: (**a**) TATB_(001)_/F2314, (**b**) TATB_(010)_/F2314 and (**c**) TATB_(100)_/F2314. The insets are enlarged views of the arrangements of successive molecular planes.

To investigate the influence of crystallographic orientation on the mechanical behavior of TATB/F2314, three low-index TATB facets, (001), (010), and (100), were considered, as shown in Figure 1. Each model consisted of an F2314 layer confined between two TATB slabs, forming a TATB/F2314/TATB sandwich structure, as shown in Figure 2a–c. The corresponding models are hereafter denoted as TATB_(001)_/F2314, TATB_(010)_/F2314, and TATB_(100)_/F2314, respectively. The insets show the stacking of successive molecular planes in TATB for each orientation. The dimensions of all sandwich models were ~130 Å, ~130 Å and ~300 Å along x, y and z directions, respectively.

The total potential energy of the TATB/F2314/TATB system was decomposed into the energies of the TATB and F2314 phases and their cross-interface interactions:

(1) $$E_{total}=E_{TATB}+E_{F2314}+E_{cross-\mathrm{inter}face}$$

Interactions within TATB were described using the potential developed by Gee et al. [13], which has been shown to reproduce the crystal structure and anisotropic mechanical response of TATB. The bonded and nonbonded interactions within F2314 were described using the COMPASS force field [14]. The attractive and repulsive components of the cross-interface interaction were described by the Morse and exponential repulsive potentials, respectively.

(2) $$\varphi_{Morse}=D_{0}(e^{-2\alpha (r-r_{0})}-2e^{-\alpha (r-r_{0})})$$

(3) $$\varphi_{Exp}=Ae^{-r/\rho }$$

here, r is the pair distance, and $D_{0}$, α, A, ρ are the potential parameters. The cross-interface energy is written [15,16,17] as:

(4) $$\begin{array}{l} E_{cross-\mathrm{inter}face}=\sum_{i\in TATB}\sum_{j\in F2314}\left\{4\lambda_{1}\left[{\left(\frac{r_{0}}{r_{ij}}\right)}^{12}-{\left(\frac{r_{0}}{r_{ij}}\right)}^{6}\right]+4\lambda_{2}\left[{\left(\frac{r_{0}}{r_{ij}+\alpha }\right)}^{12}-{\left(\frac{r_{0}}{r_{ij}+\alpha }\right)}^{6}\right]\right.\\ \left.+\lambda_{3}e^{-r_{ij}/\beta }+\lambda_{4}{\left(\frac{r_{0}}{r_{ij}}\right)}^{6}+\lambda_{5}[e^{-2\frac{r_{ij}-\gamma }{\delta }}-2e^{-\frac{r_{ij}-\gamma }{\delta }}]+\varphi_{core}(r_{ij})\varphi_{core}(r_{ij})\right\} \end{array}$$

where ij denotes an atom pair across the interface. All potential parameters are presented in [15,16], and those used for the TATB/fluoropolymer interface in this study are listed in Table S1. The force-field parameters were also validated by adhesive energies calculated from first principles. The adhesive energies were calculated using the VASP program with the GGA + vdW functional, a plane wave cutoff energy of 400 eV, and a 2 × 2 × 2 k-point. The force-field predictions agree well with the corresponding first-principles adhesion energies, as shown in Figure S1. In the LAMMPS implementation, the nonbonded interactions within TATB were described using a 12-6 Lennard-Jones potential with a cutoff of 10 Å and a Debye-screened Coulomb potential with a screening parameter of 2 Å^−1^ and a cutoff of 6 Å. The nonbonded interactions within F2314 were described using the COMPASS-type 9-6 Lennard-Jones potential with a cutoff of 10 Å. The TATB/F2314 cross-interface interactions were described using separately parameterized Morse and exponential repulsive potentials, each with a cutoff of 15 Å. No additional explicit cross-interface Coulomb interaction or long-range dispersion summation was employed. Further details of the nonbonded interaction implementation and an evaluation of the cross-interface cutoff effects are provided in the Supplementary Materials. This force-field framework has also been applied to TATB- and HMX-based energetic-material/polymer interfaces in our previous studies [16,18,19,20].

MD simulations were performed using the Large-scale Atomic/Molecular Massively Parallel Simulator (LAMMPS) [21]. To eliminate unreasonable molecular configurations, the models were first relaxed by energy minimization using the conjugate-gradient method with a force-convergence tolerance of 10^−8^ kcal/(mol·Å), followed by equilibration for 25 ps in the canonical (NVT) ensemble and 100 ps in the isothermal-isobaric (NPT) ensemble. Three-dimensional periodic boundary conditions were applied, and a time step of 0.5 fs was used. The relaxation simulations were conducted at 250, 300, 350, and 400 K. Representative total energy and pressure fluctuations are shown in Figure S2. The total energy and pressure exhibited statistically stationary fluctuations during the final stage of equilibration, suggesting that the systems had reached stable macroscopic conditions. However, such stability does not necessarily imply complete conformational equilibration of the amorphous F2314 chains. To evaluate the influence of the initial relaxation duration, additional simulations were performed using configurations obtained after 100 ps and 1 ns of NPT relaxation. As shown in Figure S3, the resulting mechanical responses were found to be closely comparable under the selected temperatures and strain rates, indicating that the principal mechanical observations are relatively insensitive to this change in the initial relaxation duration.

Thereafter, uniaxial tensile deformation was applied using a hybrid ensemble combining strain-controlled loading along the tensile axis with independent transverse pressure control, thereby approximating a uniaxial stress condition [22]. Constant engineering strain rates $\dot{\varepsilon }$ (1 × 10^9^ s^−1^, 5 × 10^9^ s^−1^, 1 × 10^10^ s^−1^, 5 × 10^10^ s^−1^, 1 × 10^11^ s^−1^) were imposed along the loading direction (z-axis) using the fix deform command, with atomic coordinates remapped affinely upon box deformation [23]. Simultaneously, the two transverse directions (x and y) were independently coupled to Nosé–Hoover barostats with a target pressure of zero, allowing the specimen to contract freely through the Poisson effect. The three directions were controlled independently without coupling. Typical stress fluctuations for the two transverse directions and the loading direction are presented in Figure S4. $\sigma_{xx}$ and $\sigma_{yy}$ oscillate around zero throughout the entire loading process, indicating that the transverse stresses were effectively relaxed.

The z-direction density profile was calculated by dividing the simulation box into uniform slabs of width Δz = 1 Å. Each atom was assigned to a slab according to its z coordinate, and the mass contributions of TATB and F2314 atoms were accumulated separately. The mass density of component *i* in each slab was calculated as: $\rho_{i}(z)=\sum m_{j}/A_{xy}\Delta z$, where $\sum m_{j}$ is the total mass of component i in the slab, and $A_{xy}$ is the cross-sectional area of the simulation box in the x-y plane. The intermixing region was characterized based on the spatial overlap between the TATB and F2314 density profiles. The local deformation gradient, $F_{i}$, was evaluated using a neighborhood cutoff of 6.0 Å. The corresponding local Green-Lagrange strain tensor was calculated as [24]: $E_{i}=\left(F_{i}^{T}F-I\right)/2$, where $F_{i}$ is the local deformation gradient tensor of atom *i*, and *I* is the second-order identity tensor. The local von Mises shear strain, $\eta_{i}$, was calculated from the strain tensor to characterize spatially heterogeneous distortional deformation. Here, the fractions of high shear strain atoms in TATB and F2314 [25], $f_{\eta }^{\alpha }=N_{\alpha }(\eta_{i}>0.2)/N_{\alpha }$, α=TATB or F2314, were calculated with $\eta_{i}$ greater than 0.2. For void analysis, the simulation box was discretized into cubic voxels with a grid spacing of 1.25 Å. A voxel was classified as vacant when its center was more than 3.0 Å from the nearest atom. Connected vacant regions with volumes exceeding 500 Å^3^ were identified as effective voids.

## 3. Results and Discussion

### 3.1. Orientation-Dependent Interfacial Structures

**Figure 3:**
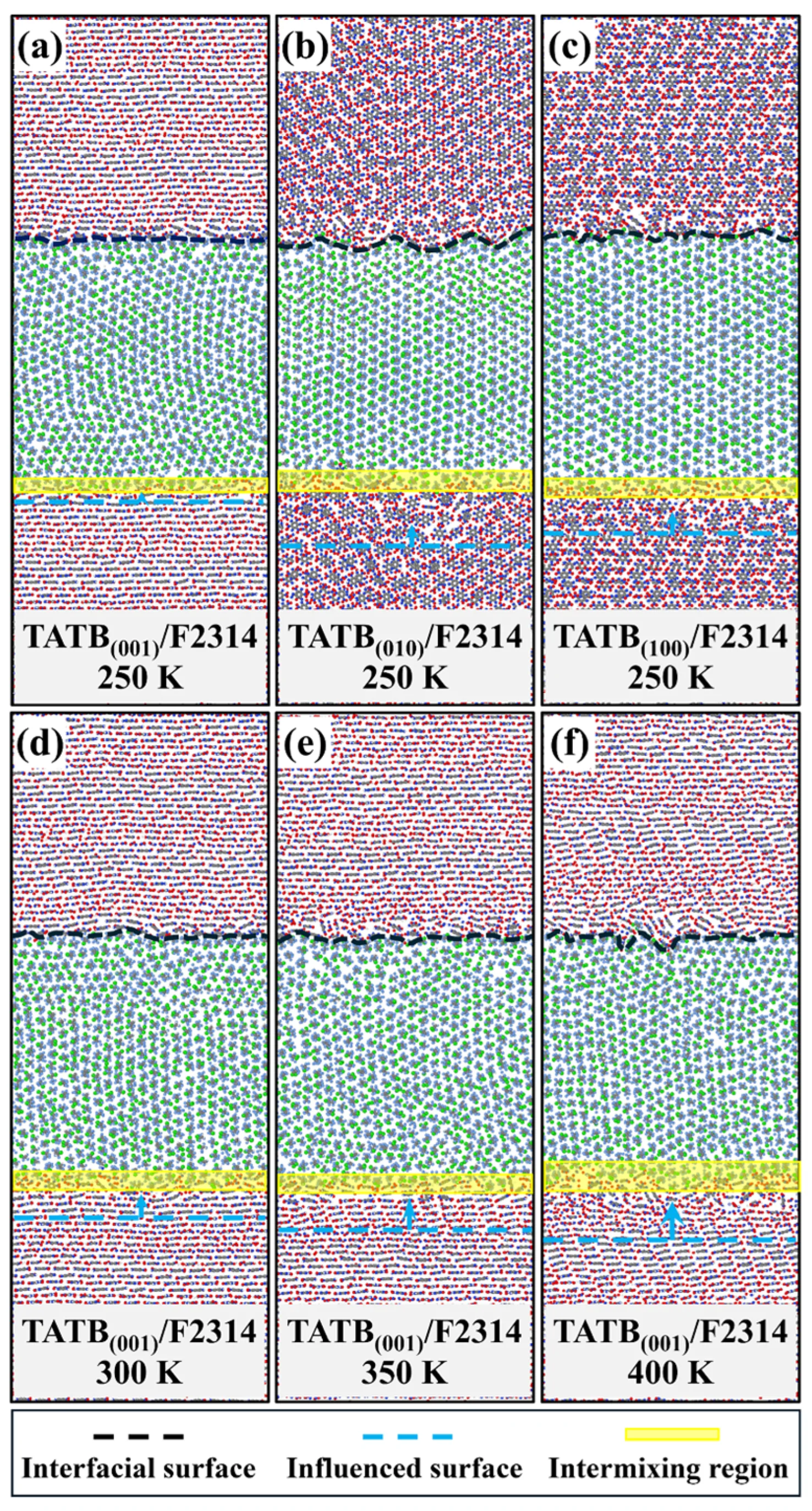
Relaxed structures at 250 K: (**a**) TATB_(001)_/F2314, (**b**) TATB_(010)_/F2314, and (**c**) TATB_(100)_/F2314. Relaxed TATB_(001)_/F2314 structures at (**d**) 300 K, (**e**) 350 K, and (**f**) 400 K. The black dashed line marks the interfacial surface, the blue dashed line marks the least disturbed TATB layer, and the yellow shading refers to the intermixing region.

The relaxed configurations of the three TATB/F2314 interfaces at 250 K are compared in Figure 3a–c. TATB_(001)_/F2314 retains a relatively planar contact feature because the flat aromatic basal planes of TATB are oriented parallel to the interfacial surfaces. Consequently, only a narrow region of the TATB phase is structurally perturbed by contact with F2314. In contrast, the (010) and (100) facets expose the edges of the TATB molecular layers and their amino and nitro substituents to the polymer. These interfacial surfaces undergo more pronounced molecular rearrangement and develop corrugated contact morphologies. The thickness of the structurally perturbed TATB region, namely the influenced region, is ~32.6 Å for TATB_(010)_/F2314 and ~21.3 Å for TATB_(100)_/F2314, compared with only ~7.6 Å for TATB_(001)_/F2314. The extent of the intermixing region follows the same order. Thus, at low temperature, the (001) basal facet forms the most regular and least intermixed contact with F2314, whereas the two edge-exposed facets produce rougher and more diffuse contact regions.

Despite its relatively ordered structure at 250 K, TATB_(001)_/F2314 exhibits pronounced thermal sensitivity. Figure 3d–f shows its relaxed configurations from 300 to 400 K. As temperature increases, the initially planar interfacial surfaces become progressively roughened, accompanied by molecular reorientation within TATB layers and an expansion of the intermixing region. These changes become particularly pronounced at 400 K, indicating that the initially regular (001) basal contacts undergo coupled structural rearrangement at the TATB-F2314 interface. More detailed analysis is provided in Figure S5.

**Figure 4:**
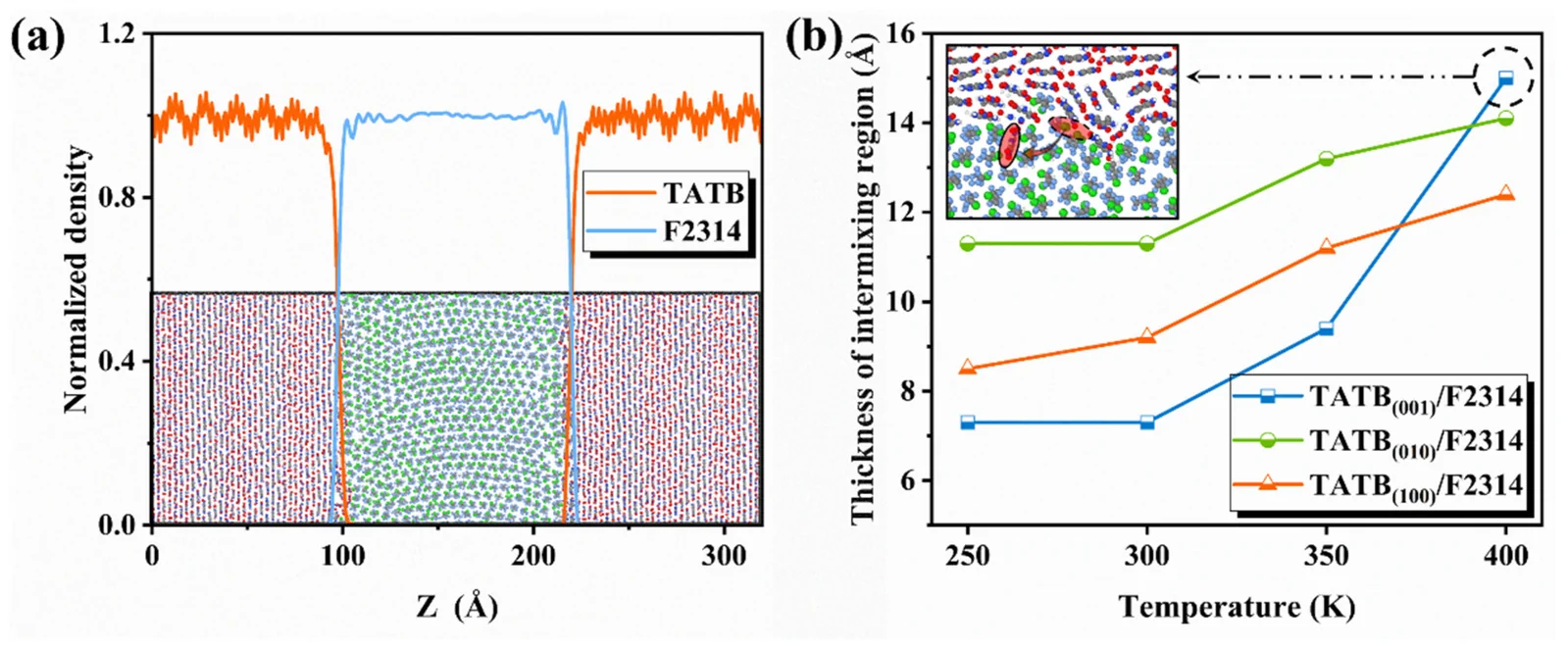
(**a**) Density profiles of TATB and F2314 along the z direction for TATB_(001)_/F2314 at 250 K. (**b**) Thicknesses of the intermixing regions in TATB_(001)_/F2314, TATB_(010)_/F2314 and TATB_(100)_/F2314 at different temperatures. The inset shows an enlarged view of the contact surface in TATB_(001)_/F2314 at 400 K.

The density profiles provide a quantitative description of this structural evolution, as shown for TATB_(001)_/F2314 at 250 K in Figure 4a. The normalized density of TATB drops sharply from its bulk value to zero on passing the interfacial surface, while that of F2314 rises to one over a short distance. Crucially, the two profiles do not switch abruptly but overlap across a finite region at the interface, where both densities remain non-zero. This overlap provides direct evidence for the coexistence of TATB and F2314 and defines the thickness of the intermixing region. The intermixing region thicknesses extracted for the three orientations at different temperatures are summarized in Figure 4b. The results reveal a pronounced coupling between orientation and temperature. At 250 K, the thicknesses of the intermixing regions follow the order TATB_(010)_/F2314 > TATB_(100)_/F2314 > TATB_(001)_/F2314, consistent with the relaxed configurations in Figure 3. The intermixing region thicknesses of the TATB_(010)_/F2314 and TATB_(100)_/F2314 increase gradually with temperature, whereas TATB_(001)_/F2314 retains the smallest one up to 350 K. At 400 K, however, the intermixing region thickness of TATB_(001)_/F2314 increases to approximately 15 Å, becoming the largest among the three systems. The inset of Figure 4b reveals local disruption of the initially planar basal contact and pronounced rotational rearrangement of the TATB molecules. This rotational rearrangement agrees with the substantially increased intermixing thickness at 400 K. Such rotational motion is not an accidental event but becomes more frequent at elevated temperatures, as shown in Figure S6. Thus, TATB_(001)_/F2314, despite being the most structurally regular and least intermixed at low temperature, exhibits the most pronounced coupled structural rearrangement at elevated temperature.

**Figure 5:**
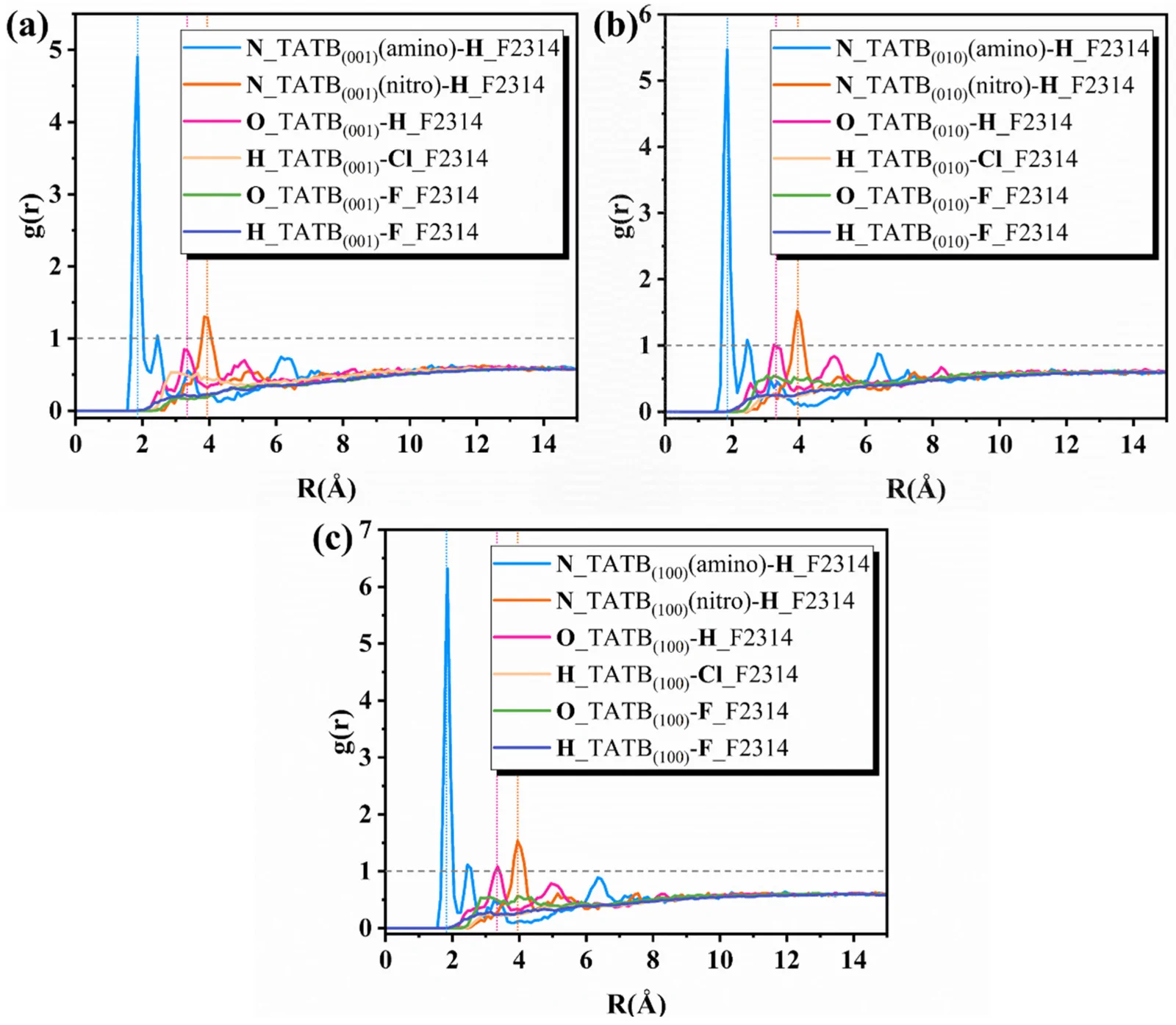
Radial distribution functions between TATB and F2314 atoms at 300 K: (**a**) TATB_(001)_/F2314; (**b**) TATB_(010)_/F2314; (**c**) TATB_(100)_/F2314. The vertical dashed lines indicate the first peaks of N_TATB(amino)-H_F2314, N_TATB(nitro)-H_F2314 and O_TATB-H_F2314 pairs.

To characterize interactions between the two phases, partial radial distribution functions (RDFs) were analyzed, as shown in Figure 5. The three N_TATB(amino)-H_F2314, N_TATB(nitro)-H_F2314 and O_TATB-H_F2314 pairs show first peaks with g(r) > 1, suggesting preferential contacts across the interfacial surfaces. Among them, the N_TATB(amino)-H_F2314 pair shows the most pronounced first peak at approximately 1.85 Å, indicating a strong structural preference for this short-range contact geometry. The first peak of O_TATB-H_F2314 is near 3.35 Å, revealing preferential intermolecular attractive contacts between the nitro oxygen and F2314 hydrogen. The N_TATB(nitro)-H_F2314 pair exhibits broader features at ~3.95 Å and ~5.0 Å, corresponding to van der Waals (vdW) and long-range spatial correlations rather than a directional hydrogen bond. The remaining three pairs show no comparably pronounced short-range preference within the first coordination region.

**Figure 6:**
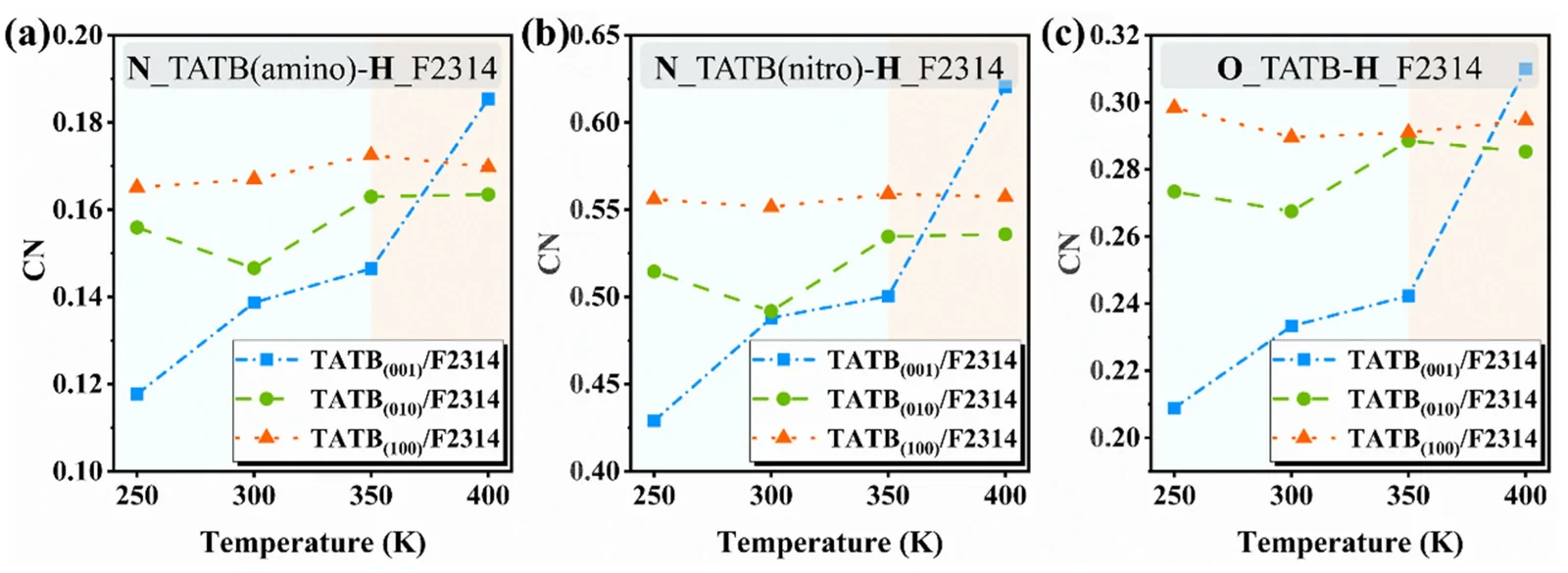
The mean interfacial coordination number values derived from the RDF results at various temperatures: (**a**) N_TATB(amino)-H_F2314 pair; (**b**) N_TATB(nitro)-H_F2314 pair; (**c**) O_TATB-H_F2314 pair.

The corresponding mean interfacial coordination number (CN) values are reported in Figure 6 based on the RDF results at different temperatures (Figure S7). For each atom pair, the first minimum of its RDF was used as the cutoff distance for identifying short-range contacts. It is noteworthy that CN values at the interface are inherently small (<1) because the analysis considers only specific atom type pairs within the geometrically confined interfacial region. Obviously, the temperature-dependent evolution of CN is strongly affected by crystallographic orientation. The CN values of TATB_(010)_/F2314 and TATB_(100)_/F2314 change only moderately between 250 and 400 K. In contrast, TATB_(001)_/F2314 shows a consistently lower CN at 250–350 K, but exhibits a pronounced increase at 400 K for all three preferential pairs. Specifically, between 350 and 400 K, the CN values of the N_TATB(amino)-H_F2314, N_TATB(nitro)-H_F2314 and O_TATB-H_F2314 pairs increase by ~26.6%, ~24% and ~27.5%, respectively. This increase can be attributed to the disruption of the initially planar basal contact, which exposes amino and nitro groups to F2314 and increases the number of intermolecular contacts.

In summary, these results show that the exposed TATB facet governs the morphology and thermal stability of the interfacial surfaces. At low temperature, TATB_(001)_/F2314 shows planar interfacial surfaces with limited intermixing, whereas TATB_(010)_/F2314 and TATB_(100)_/F2314 form more corrugated interfacial surfaces with greater intermixing. Temperature preferentially induces reconstruction of the interfacial surface in TATB_(001)_/F2314, substantially enhancing molecular intermixing and increasing the interface coordination numbers. Orientation-dependent molecular packing therefore establishes different interfacial constraints and provides distinct initial conditions for the subsequent tensile deformation.

### 3.2. Orientation-Dependent Mechanical Responses

**Figure 7:**
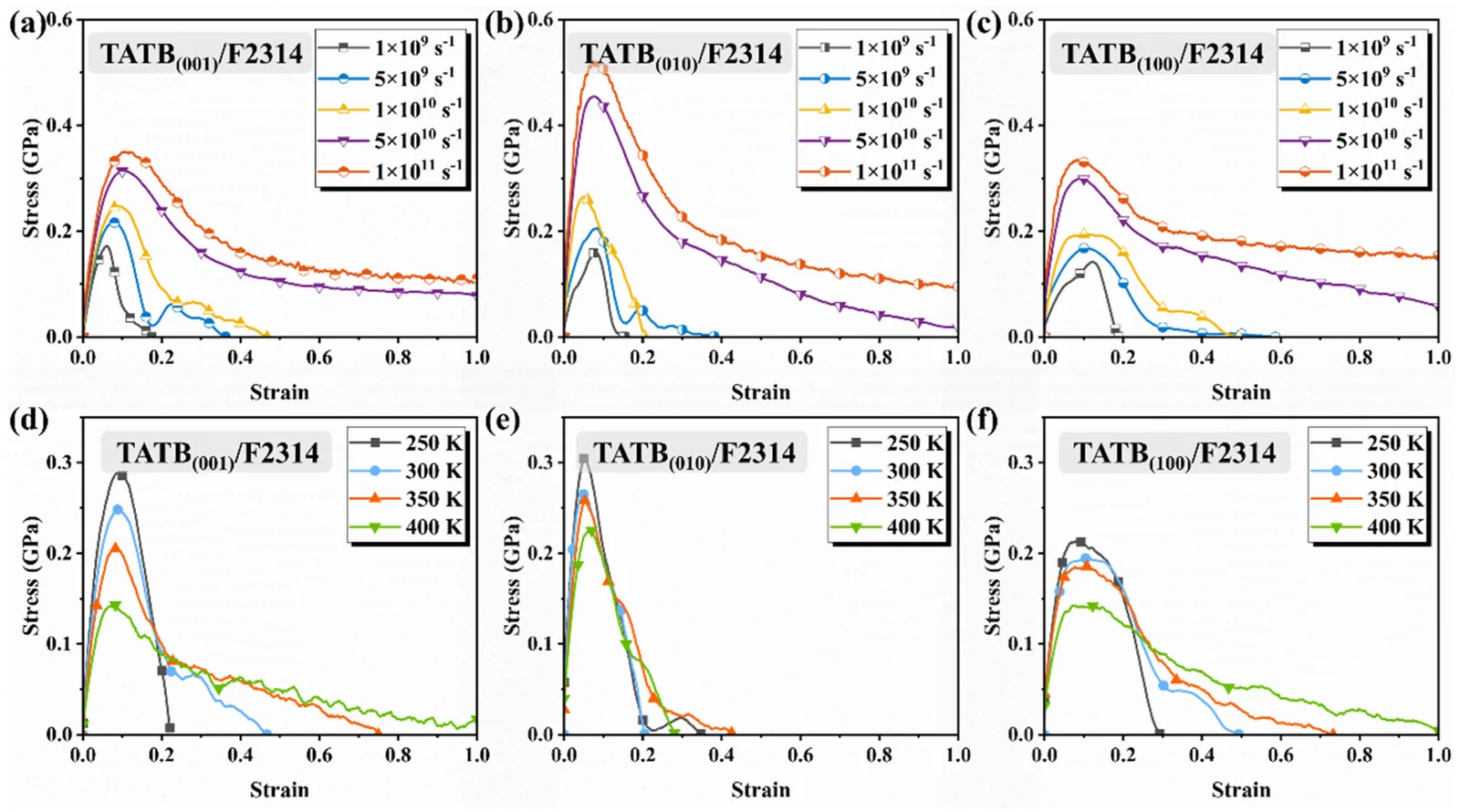
Dynamic mechanical responses of the three TATB/F2314 interface models: (**a**–**c**) Strain rate dependent responses of TATB_(001)_/F2314, TATB_(010)_/F2314, and TATB_(100)_/F2314, respectively, at 300 K and strain rates of 1 × 10^9^ s^−1^, 5 × 10^9^ s^−1^, 1 × 10^10^ s^−1^, 5 × 10^10^ s^−1^, and 1 × 10^11^ s^−1^. (**d**–**f**) Temperature-dependent responses of the interfaces at a strain rate of 1 × 10^10^ s^−1^ and temperatures of 250, 300, 350, and 400 K.

The dynamic mechanical responses of the three TATB/F2314 interfaces under representative loading conditions are exhibited in Figure 7. All of them show an initial monotonic increase in stress, followed by a pronounced peak. After reaching the maximum stress, the curves undergo a gradual decrease during post-peak softening. Note that the calculated stress is the volume averaged virial stress of the entire sandwich model. The peak stress is interpreted as the effective tensile strength of the TATB/F2314/TATB system. At 300 K (Figure 7a–c), the peak stress increases with strain rate for all three orientations, demonstrating a consistent strain rate strengthening response. Nevertheless, the magnitude of this strengthening is strongly orientation dependent. As the strain rate increases from 1 × 10^9^ s^−1^ to 1 × 10^11^ s^−1^, the peak stress of TATB_(010)_/F2314 increases from approximately 0.160 to 0.49 GPa, corresponding to an increase by a factor of 3.06. The corresponding factors for TATB_(001)_/F2314 and TATB_(100)_/F2314 are 2.37 and 1.88, respectively. In addition, the temperature-dependent responses at 1 × 10^10^ s^−1^ are displayed in Figure 7d–f. Clearly, temperature has a pronounced effect on the effective tensile peak stress of the sandwich system. Increasing the loading temperature from 250 K to 400 K significantly decreases the peak stress of TATB_(001)_/F2314 from ~0.288 GPa to ~ 0.143 GPa, representing an approximately 50% reduction. The peak stress reductions for TATB_(010)_/F2314 and TATB_(100)_/F2314 are ~27% and ~33%. These results show that crystallographic orientation affects not only the tensile response but, more importantly, the extent of thermal softening and strain rate strengthening. The following analysis therefore focuses on how crystallographic orientation and temperature affect the apparent strain rate sensitivity.

**Figure 8:**
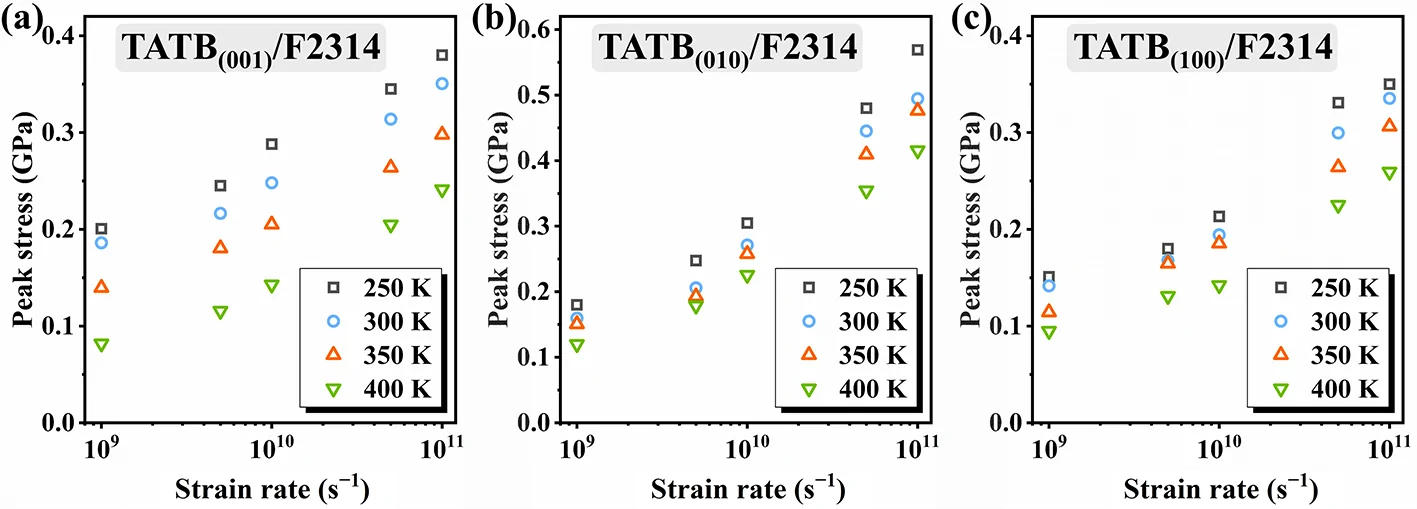
Peak stresses for (**a**) TATB_(001)_/F2314, (**b**) TATB_(010)_/F2314 and (**c**) TATB_(100)_/F2314 under different loading conditions.

To explore these coupled effects, the peak stresses obtained from all simulations are summarized in Figure 8. Again, models with different orientations exhibit obvious strain rate- and temperature-dependent mechanical responses. For a more direct comparison, the strain rate sensitivities at different temperatures are quantitatively evaluated. Usually, a power-law rate effect is assumed, with the strain rate sensitivity (SRS) coefficient, *m*, defined [26] as follows: $m=\partial \ln\sigma /\partial \ln\dot{\varepsilon }$. In this study, the apparent SRS was quantified using the peak stress and applied strain rate. Based on the mechanical responses, the apparent SRS coefficients of the three interfaces at different temperatures were calculated and are summarized in Table 1. TATB_(010)_/F2314 shows weakly temperature-dependent SRS, with its values ranging from 0.25 to 0.27 over 250–400 K. TATB_(100)_/F2314 shows a similar tendency, with values ranging from 0.20 to 0.22. Nonetheless, TATB_(001)_/F2314 displays the strongest temperature dependence, increasing from 0.14 at 250 K to 0.24 at 400 K.

These results identify two orientation-dependent features that require mechanistic explanation: the similar apparent SRS of TATB_(010)_/F2314 and TATB_(100)_/F2314 and the pronounced temperature-induced increase in the apparent SRS of TATB_(001)_/F2314. The different trends indicate that temperature and strain rate do not affect the tensile response of the three systems independently. More details are provided in the following sections.

### 3.3. Orientation-Dependent Deformation Mechanisms

**Figure 9:**
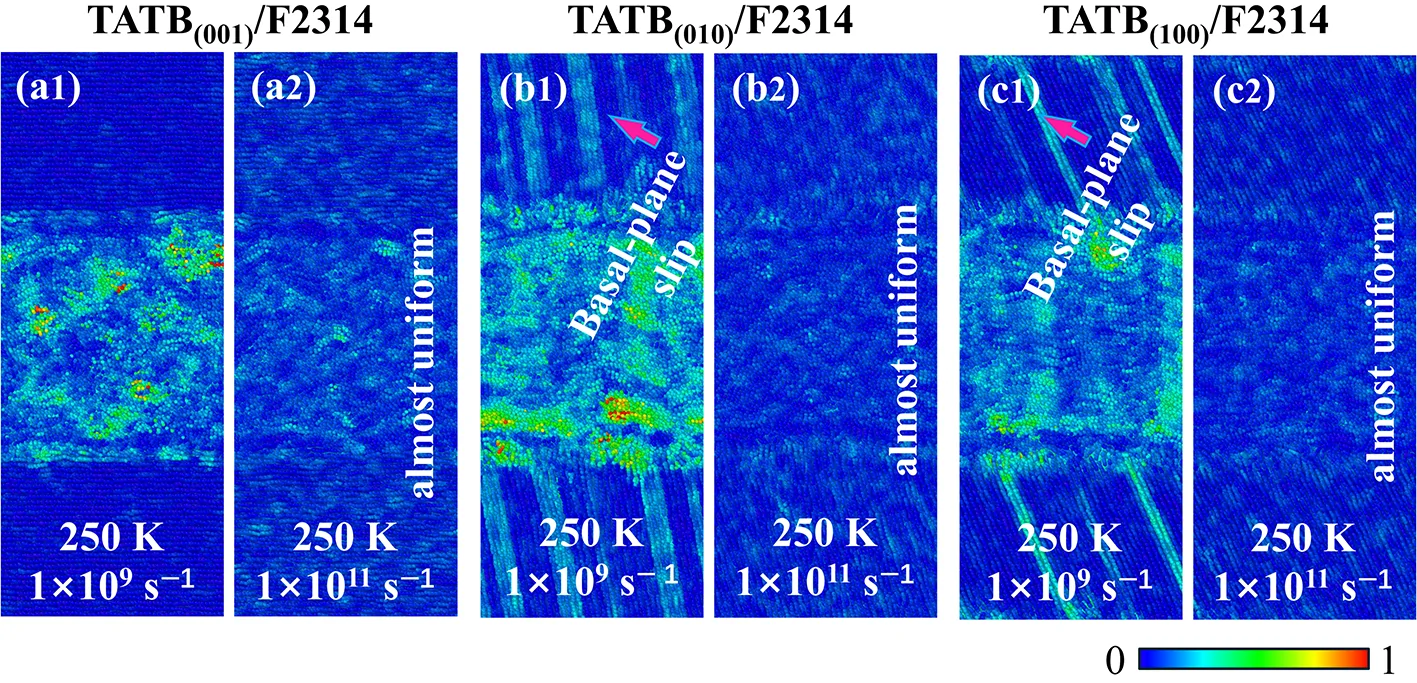
Peak-state shear strain fields for (**a1–a2**) TATB_(001)_/F2314, (**b1–b2**) TATB_(010)_/F2314, and (**c1–c2**) TATB_(100)_/F2314 at 250 K. Panels (**a1**,**b1**,**c1**) and (**a2**,**b2**,**c2**) correspond to 1 × 10^9^ s^−1^ and 1 × 10^11^ s^−1^, respectively.

Figure 9 compares the peak-state shear strain fields at 250 K and strain rates of 1 × 10^9^ s^−1^ and 1 × 10^11^ s^−1^, where Figure 9a–c correspond to TATB_(001)_/F2314, TATB_(010)_/F2314, and TATB_(100)_/F2314, respectively. Pronounced deformation heterogeneity is observed in all models, with spatially dispersed high-strain regions developing primarily in the F2314 phase. The deformation pattern within TATB, however, depends strongly on crystallographic orientation. In TATB_(001)_/F2314, no continuous band-like localization can be identified within the TATB phase, and the high strain regions remain predominantly distributed in F2314. By contrast, distinct band-like shear localization regions develop in TATB_(010)_/F2314 and TATB_(100)_/F2314 at relatively low strain rates.

Inspection of the corresponding molecular configurations shows that these high strain bands are aligned with the TATB molecular layers. Adjacent layers also slide relative to one another. This behavior is caused by the layered structure of TATB, in which strongly associated molecular layers are separated by comparatively weak van der Waals interactions [27,28]. Based on their structures and directional projection indices (Table S2) estimated by the non-affine displacements of the TATB molecular centers, the observed slip systems are identified as (001) [120] and (001) [210] slips in TATB_(010)_/F2314 and TATB_(100)_/F2314, respectively. {001}<120> has been reported to have a relatively low slip barrier and to be among the most readily activated slip systems [29]. This may explain why the dislocation-mediated basal-plane slip is visible at relatively low strain rates in TATB_(010)_/F2314 and TATB_(100)_/F2314 (Figure 9b,c). By contrast, the loading axis is almost perpendicular to the basal plane, leading to a limited shear component parallel to the basal plane, consistent with the absence of basal-plane slip. Increasing the strain rate progressively reduces the development of the slip bands in TATB_(010)_/F2314 and TATB_(100)_/F2314. At the highest strain rates, localized deformation within TATB is fully suppressed, resulting in more uniformly distributed strain fields. This behavior indicates a rate-dependent kinetic crossover in the deformation mechanism.

**Figure 10:**
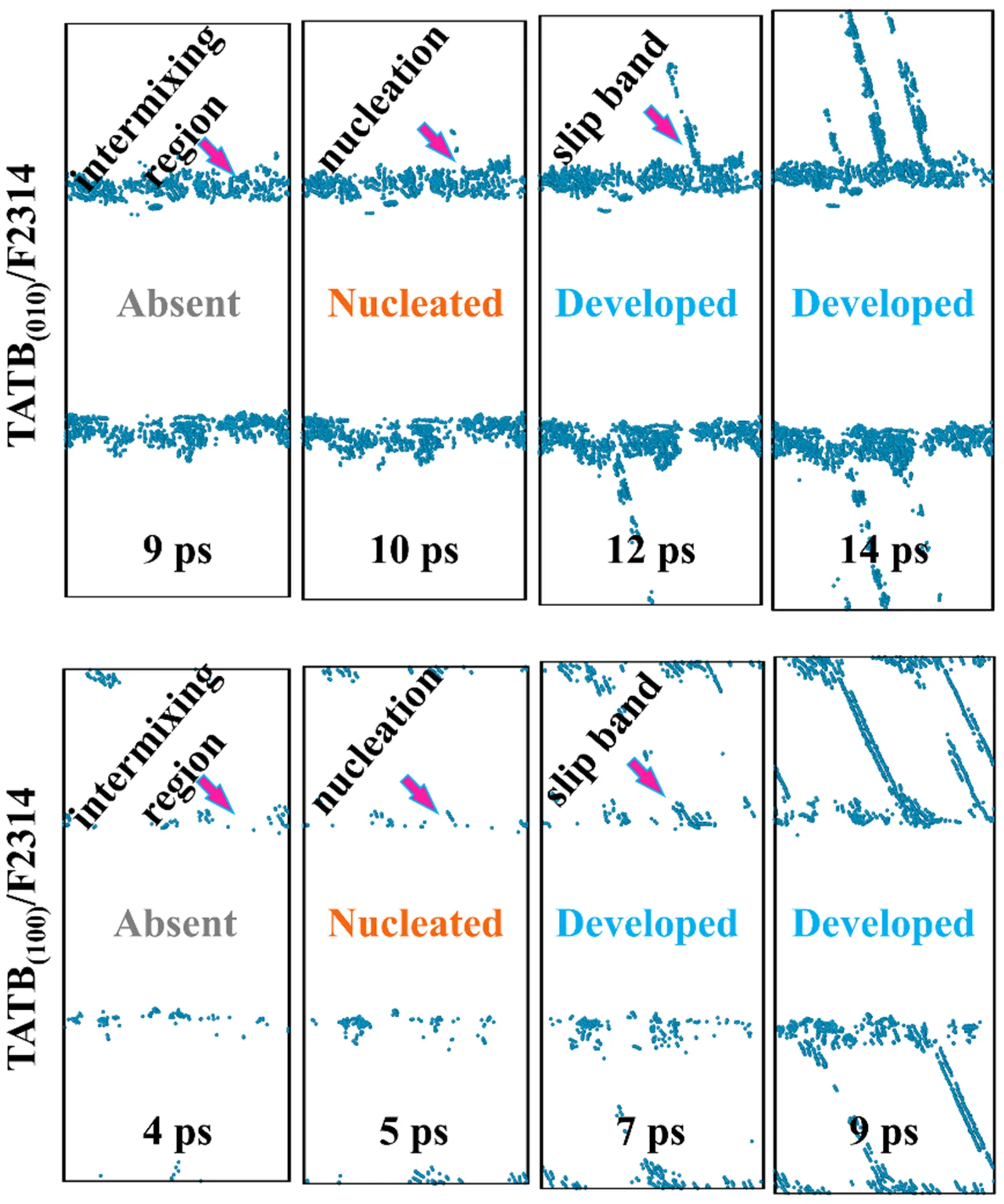
Nucleation and evolution of slip bands in TATB_(010)_/F2314 and TATB_(100)_/F2314 at a strain rate of 1 × 10^10^ s^−1^. For clarity, only TATB atoms with $\eta_{i}$ greater than 0.2 are displayed.

It is well known that the nucleation and development times are markedly different among slip systems, especially in strongly anisotropic materials. To quantitatively analyze the observed mechanism transition behavior, Figure 10 shows the nucleation and evolution of slip as a function of loading time for TATB_(010)_/F2314 and TATB_(100)_/F2314 at 250 K. The complete evolution process is provided in Figure S8. For the (010) orientation, atoms with high shear strain are primarily concentrated around the intermixing region at 9 ps because of its locally complex geometry. Local slip nucleation is observed at ~10 ps, followed by the gradual propagation of basal-plane slip across the TATB phase at approximately 12 ps. Subsequently, additional slip systems are activated, promoting further slip development. This is to say, the nucleation time of the (001) [120] slip activated in TATB_(010)_/F2314 is ~10 ps. A similar process occurs in TATB_(100)_/F2314. No slip can be seen at 4 ps, whereas local slip nucleation begins at approximately 5 ps. After that, the basal slip bands propagate and broaden, giving a nucleation time of ~5 ps for the (001) [210] slip activated in TATB_(100)_/F2314. It is also important to note that the nucleation times of both slip modes remain nearly unchanged at different strain rates (compare with Figure S9). This observation suggests that, within the examined rate range, the formation of recognizable slip structures is governed by nucleation time and is relatively insensitive to strain rate. A similar phenomenon has been reported in a recent study, in which orientation-dependent deformation mechanisms were found to evolve over markedly different timescales [30].

The slip nucleation times identified here become comparable to the available loading-time window as the strain rate approaches 1 × 10^10^ s^−1^. Below this rate, the available time is sufficiently long for molecular rearrangement and stable slip formation. Above this strain rate, substantial deformation accumulates before the characteristic slip nucleation and development processes can be completed, triggering widespread distributed molecular deformation (Figure S10) and disrupting the local structure required for stable slip nucleation and propagation. Accordingly, the lower-strain-rate range is referred to as the slip-dominated regime (regime I), while the higher-strain-rate range is characterized by distributed molecular deformation (regime II). Note that the change in deformation behavior observed near 1 × 10^10^ s^−1^ is interpreted as a kinetic crossover under the present MD conditions. To further examine the influence of the available relaxation time, an additional strain-hold relaxation simulation was performed at 250 K. Following rapid tensile loading to 10% engineering strain at 5 × 10^10^ s^−1^, no well-developed basal-plane slip bands were observed. However, recognizable slip bands subsequently emerged during a 50 ps relaxation period at the same imposed axial strain (Figure S11), accompanied by substantial stress relaxation. This result indicates that the apparent suppression of crystallographic slip during rapid loading is, at least in part, associated with the limited time available for coordinated interlayer motion. Consequently, the crossover near 1 × 10^10^ s^−1^ should be regarded as dependent on the present simulation conditions.

The thermal activation volume, V*, could be invoked to investigate changes in deformation mechanism. Considering the Arrhenius-type equation, the thermal activation volume can be written as [26]: $V*=kT\left(\partial \ln\dot{\gamma }/\partial \tau \right)$. Here, k is the Boltzmann constant, T is the absolute temperature, $\dot{\gamma }$ is the shear strain rate, τ is the shear stress. Based on this formula, the thermal activation volumes are estimated to be 349 Å^3^ and 367 Å^3^ for TATB_(010)_/F2314 and TATB_(100)_/F2314 in regime I, while the corresponding values in regime II are 166 Å^3^ and 148 Å^3^, respectively. The similar thermal activation volumes indicate that both interfaces share the same deformation mechanism within each regime, while the mechanisms operating in regimes I and II are distinct. It is worthy to note that the calculated values are smaller than those reported for other materials [31,32]. Such small thermal activation volumes therefore require further elaboration and clarification.

The physical picture describing the relationship between the thermal activation volume and the length scale of dislocation activity is [33] $V_{theory}^{*}=b_{eff}\times \xi \times l*$. The basal-plane minimum-energy paths contain several intermediate minima [34], indicating dissociation of a complete basal dislocation into multiple partial components. Based on the spacing between adjacent minima [34], an effective partial-slip displacement of $b_{eff}=2.75\pm 0.25$ Å is estimated. ξ is the dislocation swept distance during one activation event ($\xi =0.15b_{eff}$); l* is the dislocation segment length and can be geometrically assumed to be [35]: $l*\approx \rho_{dis}^{-0.5}$ ($\rho_{dis}$ is the dislocation density). Considering the reported characteristic extent of dislocation cores in TATB [34], the dislocation density is independently estimated to be ~1 × 10^15^/m^2^. And then, the theoretical activation volume is calculated to be 358–426 Å^3^. Interestingly, this theoretical value is similar to that for TATB(010)/F2314 and TATB_(100)_/F2314 in regime I, further supporting the possibility of slip mobility-dominated deformation in regime I observed in Figure 9 and Figure 10. By contrast, the thermal activation volumes estimated in regime II are even less than half of $V_{theory}^{*}$, to a certain degree, suggesting that conventional dislocation slip alone cannot account for deformation in this regime and that a more localized deformation mechanism becomes dominant. This result further demonstrates that TATB_(010)_/F2314 and TATB_(100)_/F2314 exhibit a similar rate-dependent change in the dominant deformation behavior as the strain rate increases, thereby providing a direct explanation for their comparable apparent SRSs.

**Figure 11:**
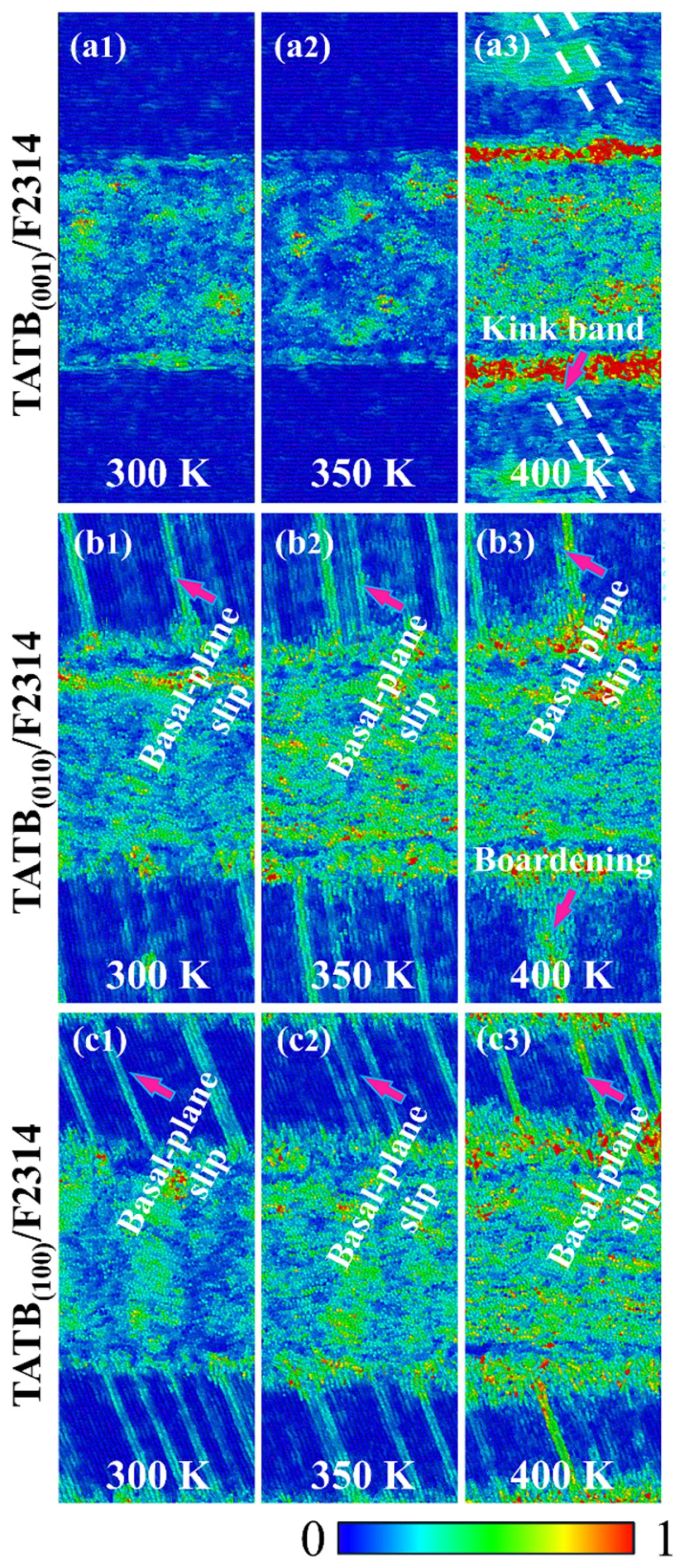
Peak-state strain fields for (**a1–a3**) TATB_(001)_/F2314, (**b1–b3**) TATB_(010)_/F2314 and (**c1–c3**) TATB_(100)_/F2314 at a strain rate of 1 × 10^9^ s^−1^. Panels 1–3 correspond to 300, 350, and 400 K, respectively.

**Figure 12:**
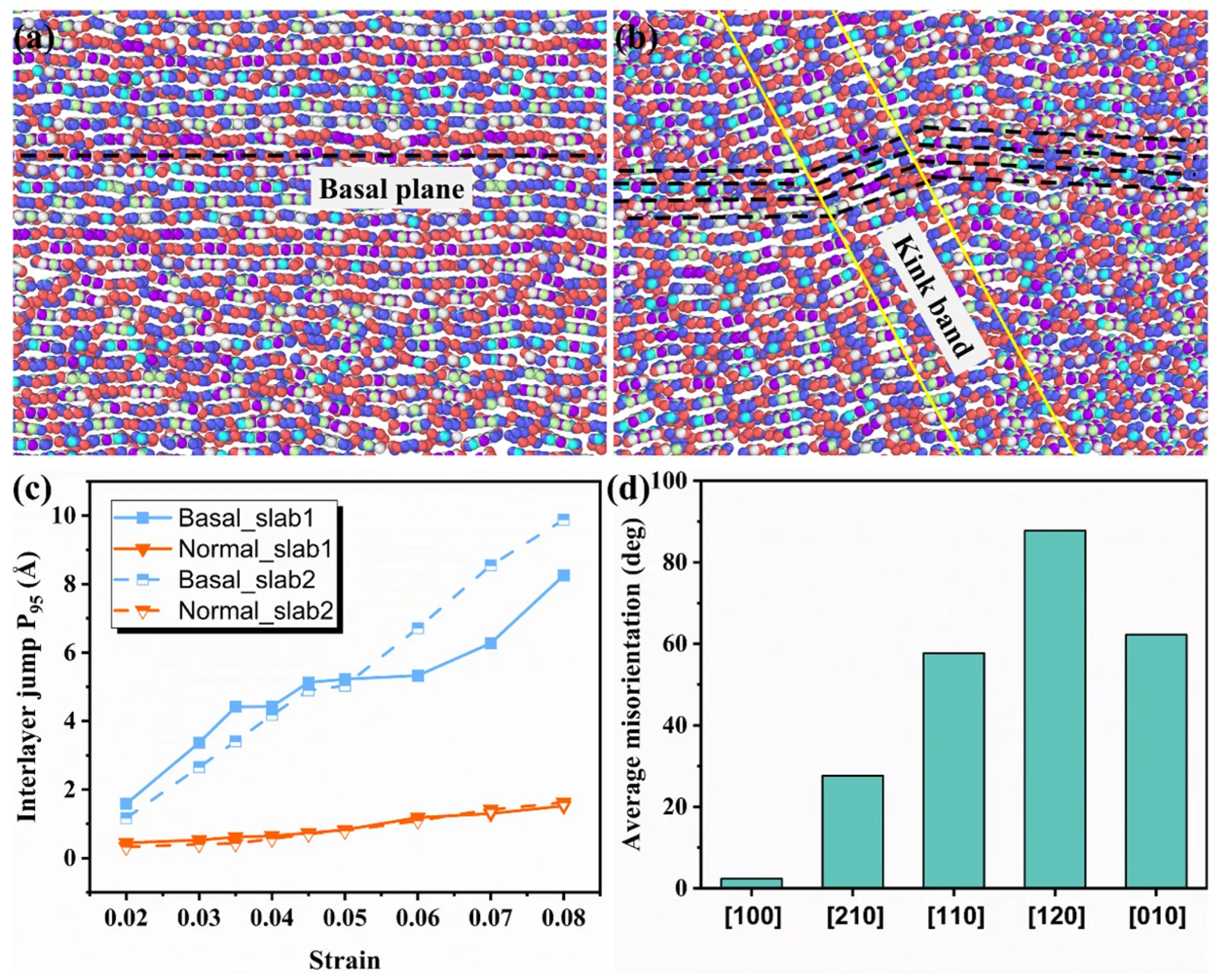
Representative molecular configurations in the (**a**) reference and (**b**) peak-stress states, respectively. (**c**) Evolution of the P_95_ (the 95th percentile) magnitudes of the basal and normal components of the relative displacement between adjacent (001) molecular layers. (**d**) Average misorientation between the principal basal interlayer-displacement direction and the candidate crystallographic directions at the peak-stress state.

Figure 11 also shows the peak-state deformation fields at 1 × 10^9^ s^−1^ as a function of temperature. Obviously, increasing temperature enhances deformation in both TATB and F2314 phases for all three orientations. The F2314 phase continues to exhibit spatially dispersed high strain regions that become progressively pronounced as temperature increases. The temperature-dependent deformation of TATB remains orientation-specific. For TATB_(001)_/F2314, deformation within TATB remains limited and no continuous shear band develops at low temperature. At 400 K, however, pronounced heterogeneous deformation appears near the reconstructed interface, accompanied by loss of molecular-layer planarity and out-of-plane distortion. Unlike the shear bands previously observed in MD simulations of TATB crystals [36,37], several adjacent (001) molecular layers undergo cooperative rotation and bending, resulting in the formation of localized kink bands (Figure 11a3). This rotation and bending can be more clearly observed by comparing with the reference-state molecular configuration (Figure 12a) and peak-stress state configuration (Figure 12b). Layer-resolved trajectory analysis further shows that kink band formation is accompanied by relative shear displacement between adjacent (001) molecular layers (Figure 12c), with the principal displacement direction close to [100] (Figure 12d). Details of the analysis method are provided in the Supplementary Materials. Coupled structural rearrangement of the initially planar interface increases local structural heterogeneity and partially relaxes the packing constraints on molecular layer rotation and interlayer shear. Together with the enhanced ability to overcome the activation barrier for kink band formation at elevated temperature, these changes facilitate kink band formation in TATB_(001)_/F2314. At 400 K, the activation of kink band mediated deformation introduces an additional rate-dependent process. At lower strain rates, sufficient time is available for kink band nucleation and propagation, enabling more local stress relaxation. At higher strain rates, the development of the kink band is kinetically restricted (Figure S12), and a higher tensile stress is consequently required to sustain deformation, accounting for the increased apparent SRS at 400 K. Such temperature-induced activation of kink-dominated deformation explains why TATB_(001)_/F2314 exhibits a temperature-dependent apparent SRS.

Different temperature-dependent responses are observed for TATB_(010)_/F2314 and TATB_(100)_/F2314. Neither of them undergoes a temperature-induced transition in its deformation mechanism. Their contact surfaces are already corrugated and relatively intermixed (Figure 3), and basal-plane slip has been activated at low temperature. Elevated temperature increases the probability of overcoming the slip barrier with thermal assistance. As a result, these basal slip bands develop further within the available time, becoming substantially broader and more numerous, as observed in Figure 11. In other words, the absence of a temperature-induced transition in deformation mechanism should hold the responsibility for the weak temperature dependence of apparent SRS in both models.

In summary, crystallographic orientation primarily determines the deformation mechanism in TATB, whereas temperature and strain rate control its activation and subsequent evolution. Basal-plane slip is readily activated in TATB_(010)_/F2314 and TATB_(100)_/F2314, even at low temperatures, and increasing temperature mainly promotes the further development of the pre-existing slip bands. By contrast, deformation in TATB_(001)_/F2314 remains strongly constrained at low temperatures, while elevated temperature activates a localized kink band. Rapid loading restricts the development of these time-dependent deformation processes, giving rise to the observed orientation- and temperature-dependent apparent strain rate sensitivities.

It is noteworthy that the strain rates employed in the present molecular dynamics simulations (10^9^–10^11^ s^−1^) are substantially higher than those typically encountered in experimental mechanical testing of TATB-based polymer-bonded explosives. For example, Blumenthal et al. [38] reported compression tests at strain rates of approximately 10^−3^–10^−1^ s^−1^ and dynamic tests at approximately 1.4 × 10^3^ s^−1^ for PBX 9502. Nevertheless, under practical handling and service conditions, PBXs may also be subjected to short-duration, high-intensity mechanical loading, including accidental impacts and shock events. Such extreme dynamic loading conditions have also been investigated through flyer-plate impact experiments on TATB-based PBXs [39]. These considerations highlight the relevance of investigating deformation mechanisms under rapid loading. The present simulation results provide atomistic insights into the crystallographic dependence of interfacial deformation and the competition between coordinated interlayer slip and distributed molecular deformation under rapid tensile loading. Note that the deformation-mode transition observed near 10^10^ s^−1^ reflects the kinetic competition within the present simulation conditions. Further experimental and multiscale investigations are needed to establish quantitative connections with lower-rate deformation and more complex impact-loading conditions.

### 3.4. Deformation Partitioning and Damage Behavior

**Figure 13:**
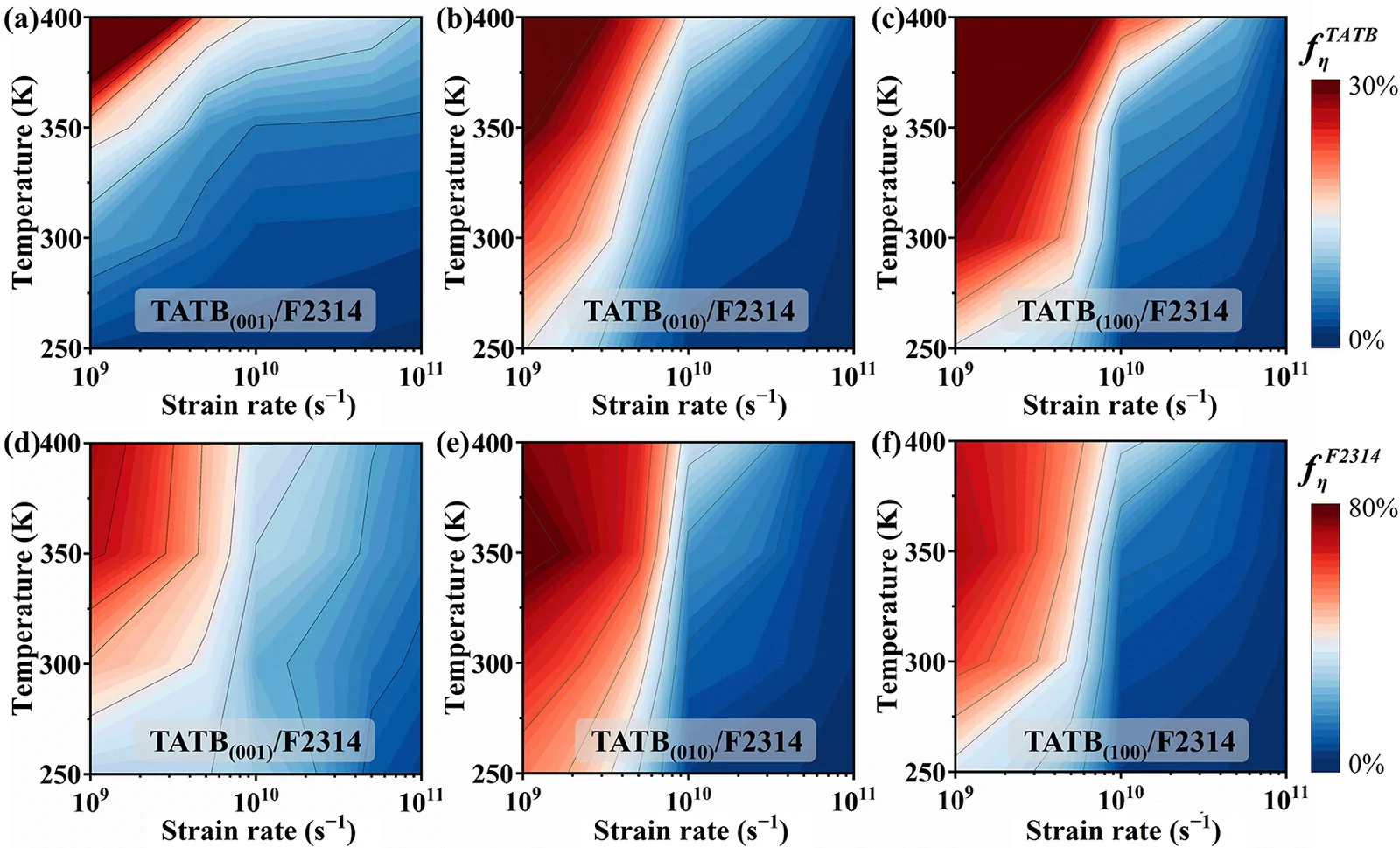
Temperature-strain rate contour maps of the peak-state deformation characteristics for TATB/F2314 interfaces: (**a**–**c**) fraction $f_{\eta }^{TATB}$ of TATB atoms with a local von Mises shear strain greater than 0.20; (**d**–**f**) fraction $f_{\eta }^{F2314}$ of F2314 atoms satisfying the same criterion.

Figure 13 further quantifies the deformation behavior of all interfaces by summarizing $f_{\eta }^{\alpha }$ (fraction of TATB or F2314 atoms with a local von Mises shear strain greater than 0.20) at peak stress. As shown in Figure 13a–c, $f_{\eta }^{TATB}$ generally increases with temperature but decreases with strain rate. For TATB_(001)_/F2314, the temperature dependence is particularly pronounced at low strain rates, where $f_{\eta }^{TATB}$ remains low at 250 K but increases substantially as the temperature approaches 400 K. This trend is consistent with the thermally activated basal-plane kinking deformation observed in Figure 11. At high strain rates, however, $f_{\eta }^{TATB}$ remains relatively low over the entire temperature range, again indicating that the shortened loading duration restricts kink band development. TATB_(010)_/F2314 and TATB_(100)_/F2314 exhibit broadly similar $f_{\eta }^{TATB}$ distributions. Both interfaces maintain relatively high values at low strain rates, followed by a marked decrease around 1 × 10^10^ s^−1^. This transition is consistent with the suppression of basal-plane slip when the imposed loading timescale becomes comparable to or shorter than its nucleation timescale. Although increasing temperature shifts the high-$f_{\eta }^{TATB}$ region toward higher strain rates, the overall contour patterns remain similar, further confirming the common strain rate-induced transition identified for the two interfaces.

Figure 13d–f shows the corresponding distributions of $f_{\eta }^{F2314}$. Similar to $f_{\eta }^{TATB}$, $f_{\eta }^{TATB}$ generally increases with temperature but decreases markedly with strain rate. Moreover, $f_{\eta }^{TATB}$ remains higher than $f_{\eta }^{TATB}$ under all investigated conditions, indicating that F2314 provides an important pathway for accommodating heterogeneous deformation. At relatively low strain rates, the longer loading duration allows segmental rotation, conformational rearrangement, and relative motion between neighboring polymer chains to develop, resulting in spatially extensive high shear regions within F2314. Increasing temperature generally promotes its deformation. The glass transition temperature (Tg) of F2314 has been reported to be ~320 K in both experimental measurements [40] and simulations [12]. Above T_g_, enhanced segmental motion facilitates conformational rearrangement and interchain sliding in F2314. Consequently, the softened F2314 phase undergoes increasingly extensive shear deformation. As the strain rate increases, deformation accumulates more rapidly than the polymer chains can reorganize in response to the evolving local stress field. The available time becomes insufficient for extensive chain rearrangement and stress relaxation, leading to the pronounced decrease in $f_{\eta }^{TATB}$ [31]. In addition, the $f_{\eta }^{TATB}$ distributions also vary among the three interfaces, although F2314 itself has no crystallographic orientation. This variation reflects the different interfacial packing, intermixing structures, and deformation mechanisms imposed by the adjacent TATB surfaces. F2314 therefore acts as a deformation accommodation phase whose molecular rearrangements are coupled to the orientation-dependent response of TATB.

**Figure 14:**
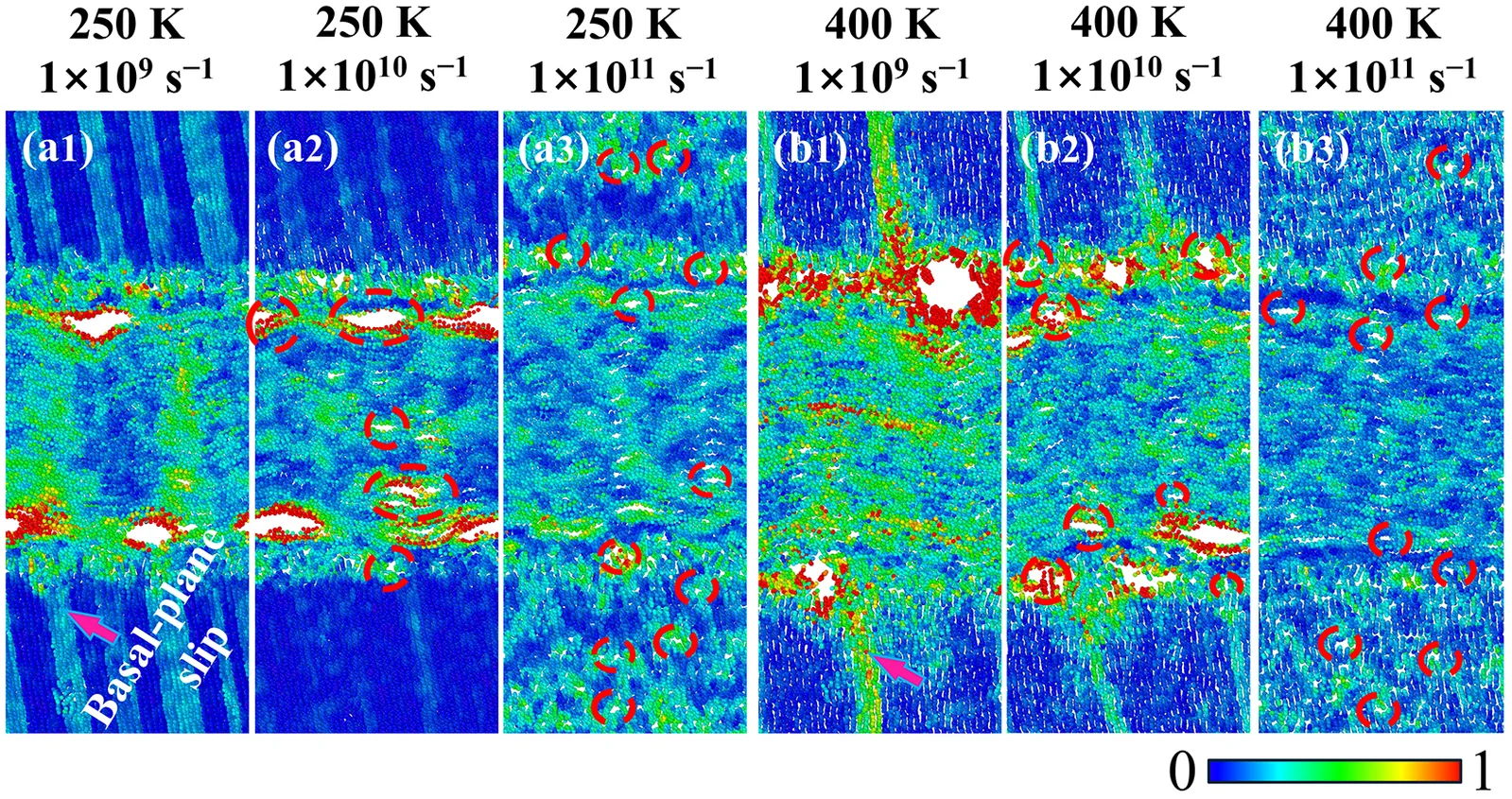
Post-peak damage morphologies of TATB_(010)_/F2314 udner (**a1**) 250 K and 1× 1^9^ s^−1^, (**a2**) 250 K and 1× 1^10^ s^−1^, (**a3**) 250 K and 1× 1^11^ s^−1^, (**b1**) 400 K and 1× 1^9^ s^−1^, (**b2**) 400 K and 1× 1^10^ s^−1^, (**b3**) 400 K and 1× 1^11^ s^−1^. All configurations correspond to the first frame on the descending branch closest to $\sigma /\sigma_{peak}\approx 0.5$. Selected voids are highlighted by red circles.

**Figure 15:**
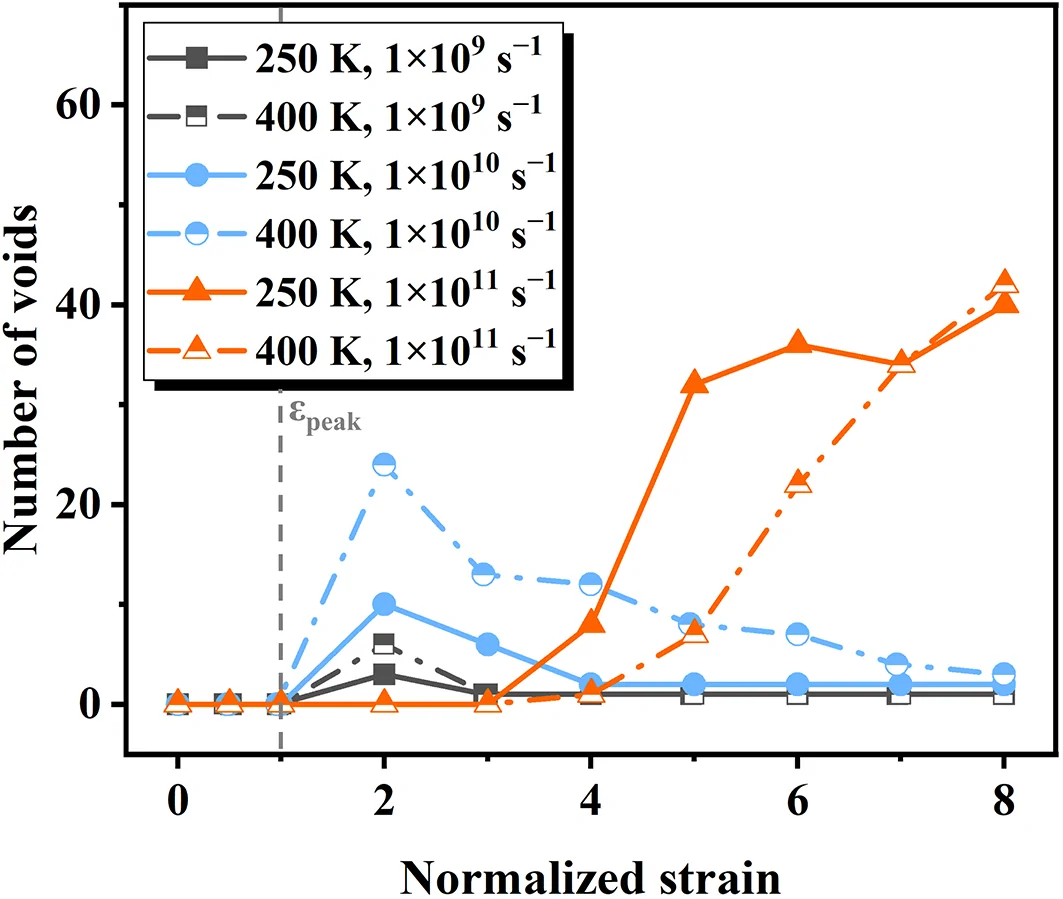
Evolution of the number of voids as a function of the normalized strain $\varepsilon /\varepsilon_{peak}$ for TATB_(010)_/F2314 at 250 and 400 K under strain rates of 1 × 10^9^ s^−1^, 1 × 10^10^ s^−1^ and 1 × 10^11^ s^−1^. The vertical dashed line indicates the peak strain.

The above results show that crystallographic orientation, temperature, and strain rate jointly influence the development of deformation mechanisms. Figure 14 further displays the typical damage mode of TATB_(010)_/F2314. A transition from localized damage at a low strain rate to multi-site distributed damage at a high strain rate is observed. Similar damage features for TATB_(001)_/F2314 and TATB_(100)_/F2314 are shown in Figure S13. At 1 × 10^9^ s^−1^, damage is strongly localized, as shown in Figure 14a1. Void nucleation occurs preferentially at or adjacent to the boundaries of the intermixing region due to local changes in molecular packing and deformation partitioning. The relatively long loading duration also allows polymer-chain rearrangement, localized deformation within TATB, and stress redistribution near the interface to develop. Consequently, the deformation is concentrated within a limited region, promoting localized void growth and coalescence. This is supported by the evolution of void number, as shown in Figure 15. At 1 × 10^9^ s^−1^, the number of voids increases modestly after the peak strain and subsequently decreases to one, indicating that the initially nucleated voids coalesce into a single larger cavity. At the intermediate strain rate of 1 × 10^10^ s^−1^, the damage morphology exhibits a transitional character. Void nucleation sites begin to extend into the TATB phase (Figure 14) and multiple voids nucleate shortly after the peak strain (Figure 15), producing an early maximum in the number of voids, particularly at 400 K. With continued loading, the number of voids gradually decreases to approximately two, suggesting that void coalescence remains operative. Compared with the response at 1 × 10^9^ s^−1^, however, damage initiation is less localized and involves a larger number of nucleation sites. This behavior indicates increasing competition between distributed void nucleation and subsequent coalescence as the strain rate increases. A distinctly different damage morphology is observed at 1 × 10^11^ s^−1^. Instead of a dominant localized void, multi-site distributed damage is observed, with multiple small, spatially separated voids developing within the interfacial region and the adjacent TATB and F2314 phases. Under rapid loading, the time available for molecular rearrangement, localized shear development, and subsequent void growth is substantially reduced. Local damage can therefore initiate at several heterogeneous sites before any individual void has sufficient time to grow and dominate the failure process. Thus, the number of voids exhibits an almost monotonic increase with strain, approaching ~40 at 8$\varepsilon_{peak}$ (Figure 15).

Temperature modifies the extent of post-peak deformation but does not fundamentally change this rate dependent failure mode (Figure 14 and Figure 15). Elevated temperature increases molecular mobility and facilitates local structural rearrangement, which can modify the size and detailed location of the damaged regions. Nevertheless, within the investigated conditions, the strain rate remains the primary factor determining whether damage develops through localized void growth or distributed void nucleation.

The damage morphologies are therefore consistent with the deformation mechanisms discussed in Section 3.3. When sufficient loading time is available, crystallographically preferred molecular rearrangement and localized shear can develop, redistributing stress toward a limited damage region. Once the loading time is shortened at a high strain rate, both coordinated deformation and void growth are restricted, resulting in multi-site distributed damage.

## 4. Conclusions

The effects of crystallographic orientation, temperature, and strain rate on the interfacial structure and deformation behavior of TATB/F2314 were investigated by molecular dynamics simulations. The main conclusions are summarized as follows.

(1) Crystallographic orientation strongly affects the relaxed structures. At low temperatures, TATB_(001)_/F2314 forms a planar basal contact with limited intermixing, whereas TATB_(010)_/F2314 and TATB_(100)_/F2314 exhibit more corrugated interfacial surfaces with greater intermixing. Nevertheless, the interfacial surfaces in TATB_(001)_/F2314 exhibit the greatest temperature sensitivity and undergo pronounced reconstruction at 400 K.
(2) All three systems exhibit strain rate strengthening and thermal softening, while the magnitude of these effects depends on crystallographic orientation. TATB_(010)_/F2314 and TATB_(100)_/F2314 show weakly temperature-dependent apparent strain rate sensitivities, whereas TATB_(001)_/F2314 exhibits the most pronounced temperature dependence.
(3) Basal-plane slip is activated in TATB_(010)_/F2314 and TATB_(100)_/F2314 at relatively low strain rates, with dominant slip directions of (001) [120] and (001) [210], respectively. Increasing the strain rate shortens the available loading time and suppresses stable slip band development, resulting in a transition toward distributed molecular deformation. In TATB_(001)_/F2314, coupled structural rearrangement facilitates kink band formation accompanied by [100] dominated interlayer shear.
(4) F2314 provides an important pathway for heterogeneous deformation and stress redistribution. With increasing strain rate, the limited time available for coordinated molecular deformation and void growth shifts the damage mode from localized void growth and coalescence near the intermixing region to multi-site distributed damage across the interfacial region and the adjacent TATB and F2314 phases.

Overall, crystallographic orientation determines the accessible deformation pathways, whereas temperature and strain rate regulate their activation and evolution. Nevertheless, the characteristic timescale and kinetic crossover range under the present simulation conditions should be determined more precisely, and the quantitative contributions of TATB and F2314 to the strain rate sensitivity remain to be clarified in future work.

## Figures and Tables

**Table 1:** Apparent strain-rate sensitivity (*m*) of the three TATB/F2314 interfaces.

Model	250 K	300 K	350 K	400 K
TATB_(001)_/F2314	0.14	0.14	0.16	0.24
TATB_(010)_/F2314	0.25	0.26	0.26	0.27
TATB_(100)_/F2314	0.20	0.20	0.21	0.22

## Data Availability

The raw data supporting the conclusions of this article will be made available by the authors on request.

## Supplementary Materials

The following supporting information can be downloaded at: https://www.mdpi.com/article/10.3390/polym18212692/s1, Figure S1. Adhesive energy curves across interfaces for TATB/polymer. The scatter symbols are first-principles results, and the lines are recalculated energy curves via the force-fields, Figure S2. The total energy and pressure fluctuations of TATB_(001)_/F2314 at 300 K, Figure S3. Comparison of the mechanical responses of TATB(100)/F2314 models after 100 ps and 1 ns of NPT relaxation. (a) Stress–strain curves at 250 K under strain rates of 5 × 10^9^ and 1 × 10^11^ s^−1^; (b) Representative shear strain fields at selected strain levels for the two relaxation durations. The colors represent the shear strain, from 0 to 1. Figure S4. Stress fluctuations along the x, y and z axes for TATB_(001)_/F2314 at 300 K under a strain rate of 1 × 10^11^ s^−1^, Figure S5. (a) Enlarged view of the TATB(001)/F2314 interface after relaxation at 400 K with position-restrained F2314; (b) Statistical results of the mean molecular reorientation angle of interfacial TATB molecules relative to their initial orientations and the fraction of molecules exhibiting reorientation angles greater than 15°, Figure S6. Enlarged view of the interfacial surface for the relaxed TATB_(001)_/F2314 at 500 K. Dashed red ellipses represent the positions of TATB molecules before rotation, and solid red ellipses represent their positions after rotation, Figure S7. The radial distribution functions between TATB and F2314 atoms at different temperatures: (a) TATB_(001)_/F2314; (b) TATB_(010)_/F2314; (c) TATB_(100)_/F2314. Within each row, panels 1–3 correspond to 250, 350, and 400 K, respectively, Figure S8. The nucleation and evolution of slip bands in TATB_(010)_/F2314 and TATB_(100)_/F2314 at a strain rate of 1 × 10^10^ s^−1^. For clarity, TATB atoms with $\eta_{i}$ greater than 0.2 are displayed, Figure S9. The nucleation and evolution of slip bands in TATB_(010)_/F2314 and TATB_(100)_/F2314 at a strain rate of 5 × 10^9^ s^−1^. For clarity, TATB atoms with $\eta_{i}$ greater than 0.2 are displayed, Figure S10. Peak-state shear strain fields for (a) TATB_(010)_/F2314 and (b) TATB_(100)_/F2314 at 5 × 10^10^ s^−1^ and 250 K, Figure S11. (a) Evolution of the TATB(100)/F2314 interfacial structure during strain-hold relaxation. The color scale represents the shear strain ranging from 0 to 1; (b) fraction of TATB atoms exhibiting a local von Mises shear strain greater than 0.20. The red dashed line indicates the end of the loading stage, Figure S12. Peak-state shear strain fields of TATB_(001)_/F2314 at 400 K under strain rates of (a) 5 × 10^10^ s^−1^ and (b) 1 × 10^11^ s^−1^, Figure S13. Post-peak damage morphologies of (a1–b3) TATB_(001)_/F2314 and (c1–d3) TATB_(100)_/F2314. All configurations correspond to the first frame on the descending branch closest to $\sigma /\sigma_{peak}\approx 0.5$. Atoms are colored according to the local von Mises shear strain and partial voids are highlighted by red circles. Different color scales are used to improve the visibility of local deformation: 0–1 for the 250 K panels and 0–2 for the 400 K panels, Table S1. Potential Parameters of the TATB/fluoropolymer interface, Table S2. Directional projection indices along the [120] and [210] directions.

## DURC Statement

Current research is limited to the solid mechanics and mesoscale damage evolution of polymer-bonded composites under dynamic loading, which is beneficial for revealing the fundamental failure mechanism of PBX composites, supporting safety assessment of energetic materials under impact conditions and providing basic data for material structure optimization, and does not pose a threat to public health or national security. Authors acknowledge the dual use potential of the research involving PBX composites and confirm that all necessary precautions have been taken to prevent potential misuse. As an ethical responsibility, authors strictly adhere to relevant national and international laws about DURC. Authors advocate for responsible deployment, ethical considerations, regulatory compliance, and transparent reporting to mitigate misuse risks and foster beneficial outcomes.
