# Supplementary material for: Crystallographic Origins of Temperature-Dependent Strain Rate Sensitivity and Deformation Mechanism Transitions at TATB/F2314 Interfaces: A Molecular Dynamics Study
Source: Polymers (Basel). 2026 Oct 30;18(21):2692. doi: 10.3390/polym18212692 (PMC13766055; doi:10.3390/polym18212692)
Supplement: Supplementary file 1 [file polymers-18-02692-s001.zip › polymers-4639453-Supplementary Material.pdf]

Force-Field Calculations for the TATB/Fluoropolymer Interface

The interfacial distance x in the TATB/polymer model is defined as the distance between the center of mass of TATB and the central axis of the polymer chain. The adhesion energy E as a function of x is calculated as follows:

$$E(x) = E_{total}(x) - E_1 - E_2 \quad (S1)$$

where E_{total} is the total energy of the system, E_1 and E_2 are the energies of the TATB and fluoropolymer species. The adhesive energies were calculated using the VASP program for interfacial distances x ranging from 1.8 to 10 Å within the GGA+vdW framework. The projector augmented-wave (PAW) method and the optB86b-vdW functional were employed, with a plane wave cutoff energy of 400 eV and an electronic self-consistency convergence criterion of 10^{-6} eV. No additional explicit cross-interface Coulomb interaction or long-range dispersion summation was introduced. For comparison, the adhesion energies calculated using the interfacial force field are also plotted as smooth curves in Figure S1. The force-field predictions agree well with the first-principles results, validating the force-field parameters developed for the TATB/fluoropolymer interface.

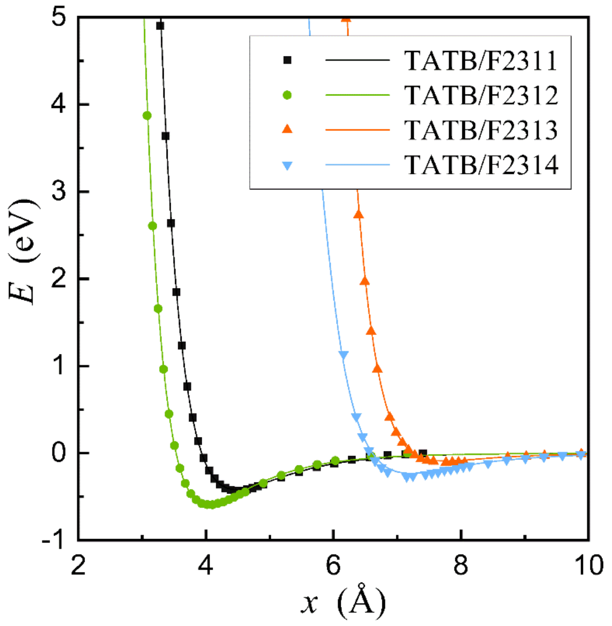

Figure S1. Adhesive energy curves across interfaces for TATB/polymer. The scatter symbols are first-principles results, and the lines are recalculated energy curves via the force-fields [16].

Implementation and Cutoff Treatment of Nonbonded Interactions

The nonbonded interactions in the TATB/F2314 composite were implemented in LAMMPS using a hybrid/overlay pair-potential framework. Within the TATB phase, the nonbonded interactions were represented by a 12-6 Lennard-Jones potential with a cutoff of 10 Å and a Debye-screened Coulomb potential with a screening parameter of 2 $Å^{-1}$ and a cutoff of 6 Å. Within the F2314 phase, the nonbonded interactions were described using the COMPASS-type 9-6 Lennard-Jones potential with a cutoff of 10 Å.

The interactions between TATB and F2314 were described using the dedicated interfacial force field developed by Long and Chen. Depending on the atomic pair types, Morse or exponential repulsive potentials were employed, with their parameters listed in Table S1. The exponential repulsive interactions were implemented using the Buckingham potential with the attractive inverse-sixth-power coefficient set to zero. All cross-interface pair interactions were assigned a cutoff distance of 15 Å. No additional explicit Coulomb interaction was applied between TATB and F2314, and no long-range electrostatic or dispersion summation was employed.

To assess possible energy and force discontinuities arising from the finite cutoff, the residual potential energy and force of each cross-interface atomic pair interaction were evaluated analytically at the cutoff distance of 15 Å. Among all cross-interface atomic pair types, the maximum absolute residual potential energy was approximately 1.05×10^{-5} kcal/mol per atomic pair, and the maximum absolute residual force was approximately 9.17×10^{-6} kcal/(mol·Å). These results indicate that the residual energy and force discontinuities of the cross-interface pair potentials are small at the selected cutoff distance.

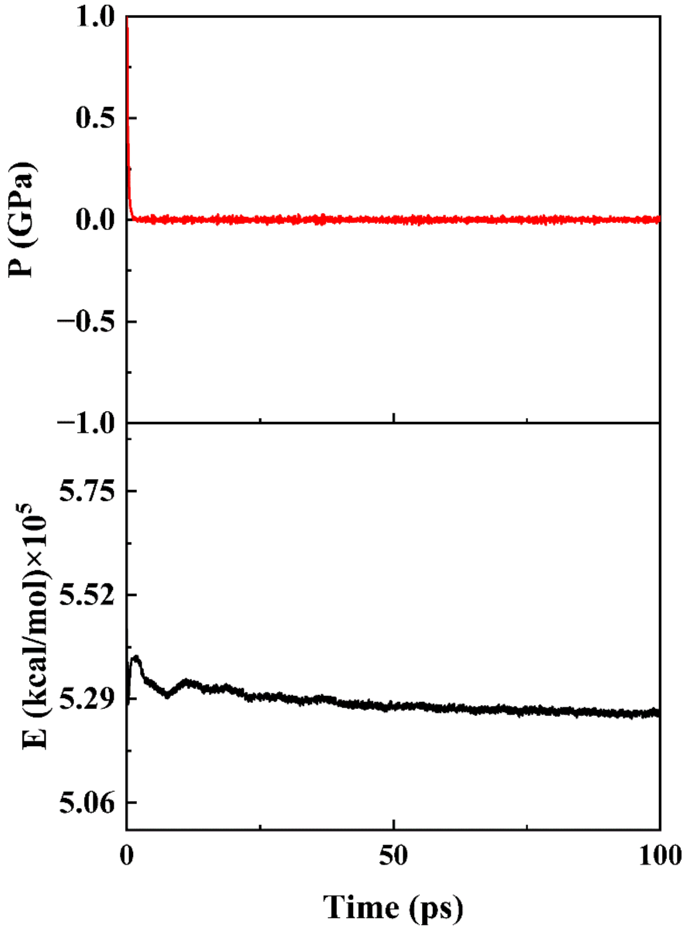

Figure S2. The total energy and pressure fluctuations of $TATB_{(001)}$/F2314 at 300 K.

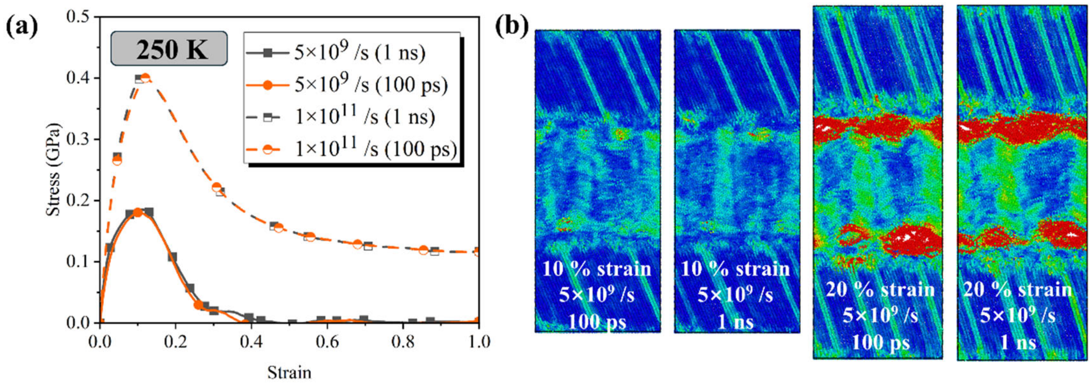

Figure S3. Comparison of the mechanical responses of TATB$_{(100)}$/F2314 models after 100 ps and 1 ns of NPT relaxation. (a) Stress-strain curves at 250 K under strain rates of 5×10^9 and 1×10^{11} s^{-1}; (b) Representative shear strain fields at selected strain levels for the two relaxation durations. The colors represent the shear strain, from 0 to 1.

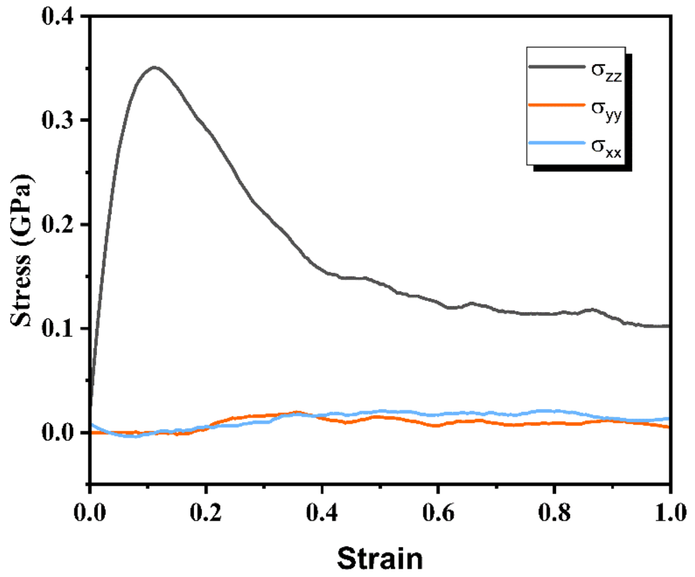

Figure S4. Stress fluctuations along the x, y and z axes for $TATB_{(001)}$/F2314 at 300 K under a strain rate of 1×10^{11} s^{-1}.

Effect of F2314 Mobility Constraints on Interfacial Structural Rearrangement

To investigate the influence of F2314 on the structural rearrangement of the $TATB_{(001)}$/F2314 interface at 400 K, additional unloaded control simulations were performed by comparing systems with freely moving and position-restrained F2314. Both systems were initialized from the same configuration. The reorientation of interfacial TATB molecules was characterized by comparing their molecular directors with the initial orientations. To reduce thermal fluctuations, the molecular directors were averaged over the 250-300 ps interval. As shown in Figure S5, restricting F2314 motion significantly reduced the reorientation of interfacial TATB molecules. The mean reorientation angle decreased from 8.79° in the freely moving F2314 system to 3.15° in the position-restrained system. Similarly, the fraction of interfacial TATB molecules exhibiting reorientation angles greater than 15° decreased from 17.48% to 2.89%. These results suggest that the structural rearrangement of interfacial TATB molecules at 400 K is influenced by the surrounding F2314. This dependence indicates that the structural evolution of TATB is coupled to the polymer environment through interfacial interactions and mechanical constraints. Therefore, the observed behavior is described as coupled structural rearrangement at the TATB/F2314 interface.

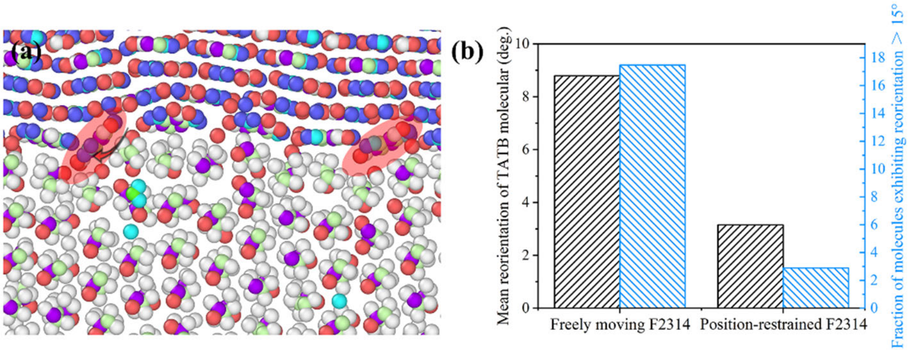

Figure S5. (a) Enlarged view of the TATB(001)/F2314 interface after relaxation at 400 K with position-restrained F2314; (b) Statistical results of the mean molecular reorientation angle of interfacial TATB molecules relative to their initial orientations and the fraction of molecules exhibiting reorientation angles greater than 15°.

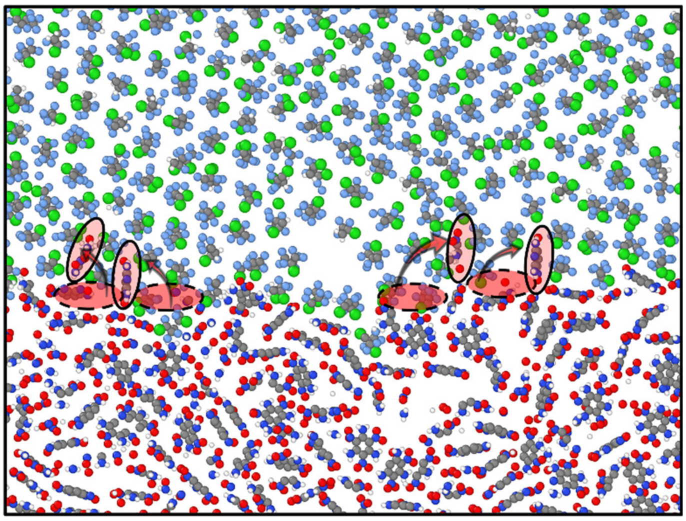

Figure S6. Enlarged view of the interfacial surface for the relaxed $TATB_{(001)}$/F2314 at 500 K. Dashed red ellipses represent the positions of TATB molecules before rotation, and solid red ellipses represent their positions after rotation.

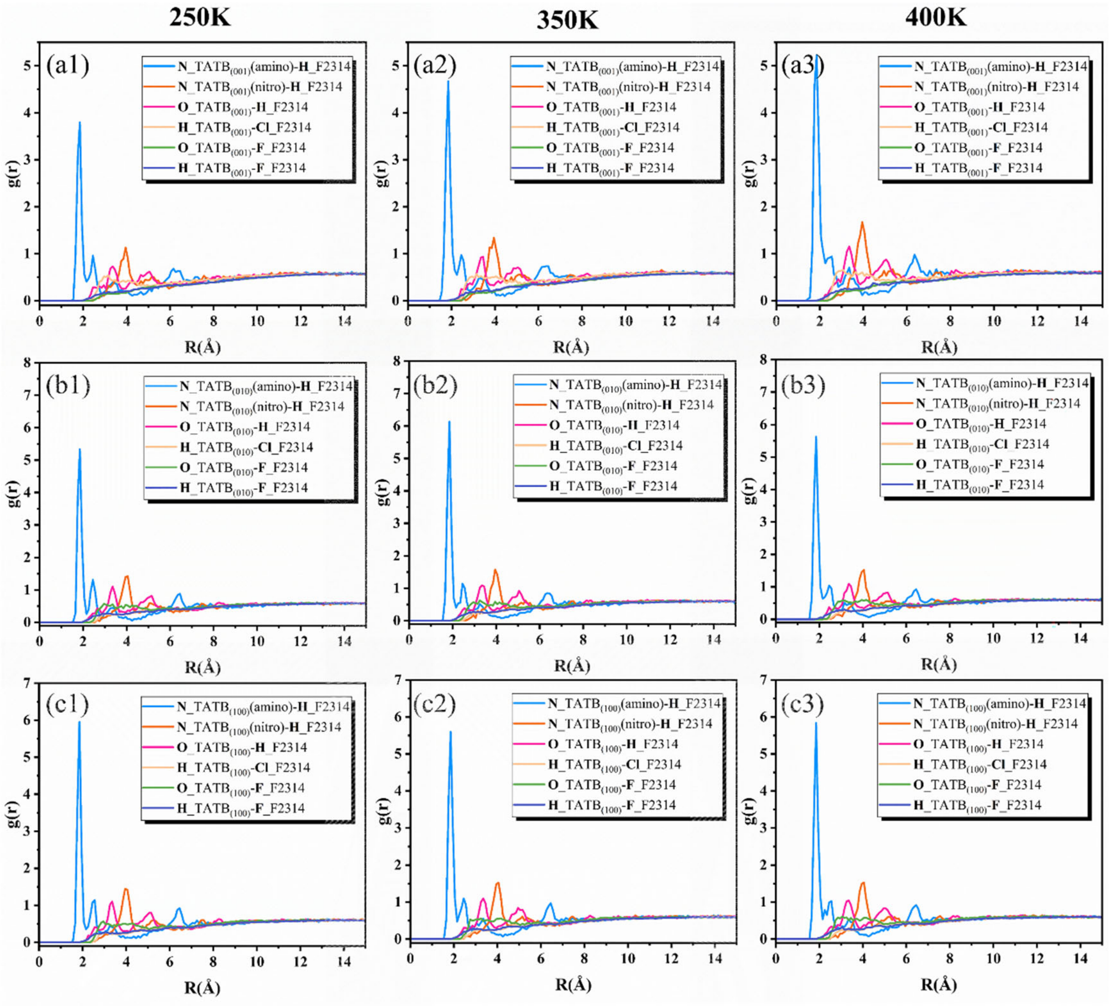

Figure S7. The radial distribution functions between TATB and F2314 atoms at different temperatures: (a) $TATB_{(001)}$/F2314; (b) $TATB_{(010)}$/F2314; (c) $TATB_{(100)}$/F2314. Within each row, panels 1-3 correspond to 250, 350, and 400 K, respectively.

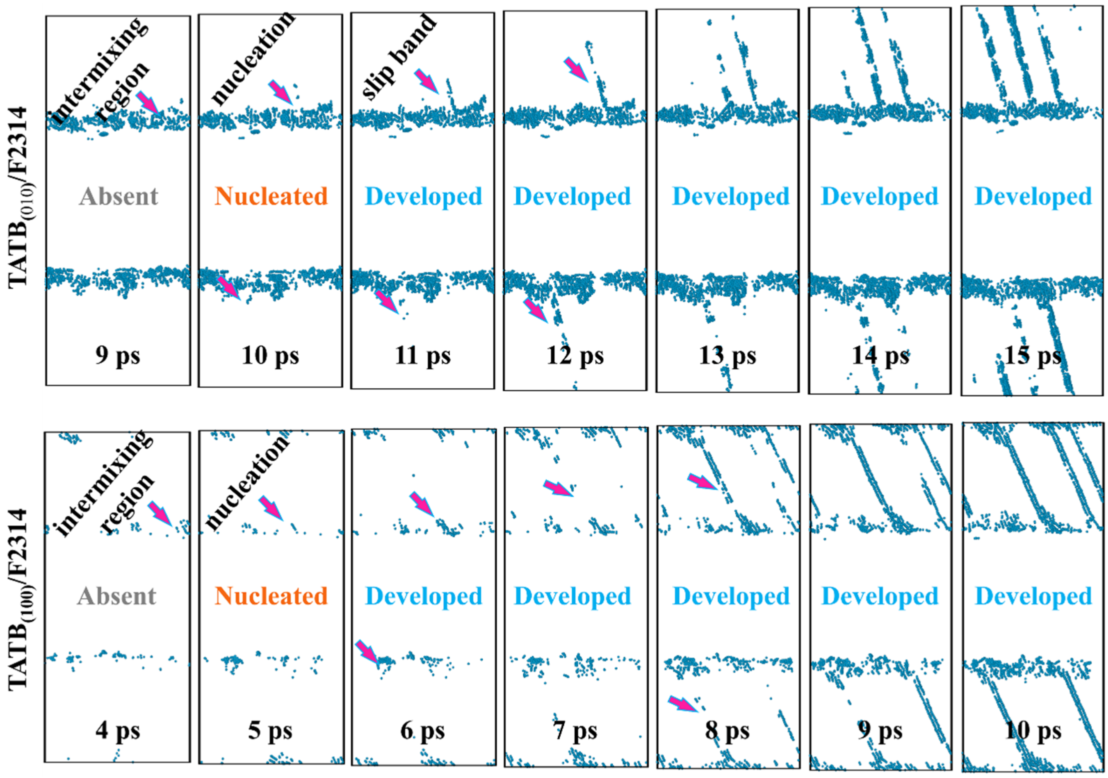

Figure S8. The nucleation and evolution of slip bands in TATB$_{(010)}$/F2314 and TATB$_{(100)}$/F2314 at a strain rate of 1×10^{10} s^{-1}. For clarity, TATB atoms with η_i greater than 0.2 are displayed.

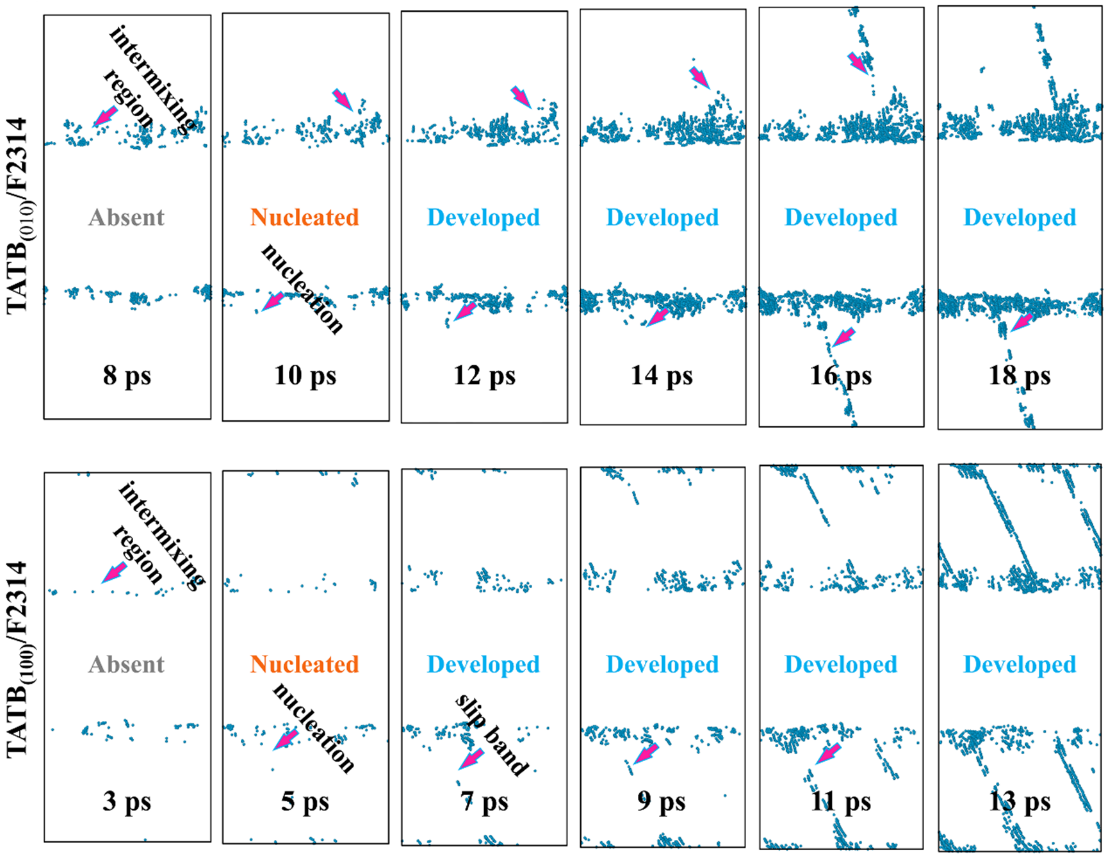

Figure S9. The nucleation and evolution of slip bands in $TATB_{(010)}$/F2314 and $TATB_{(100)}$/F2314 at a strain rate of 5×10^{9} s^{-1}. For clarity, TATB atoms with η_i greater than 0.2 are displayed.

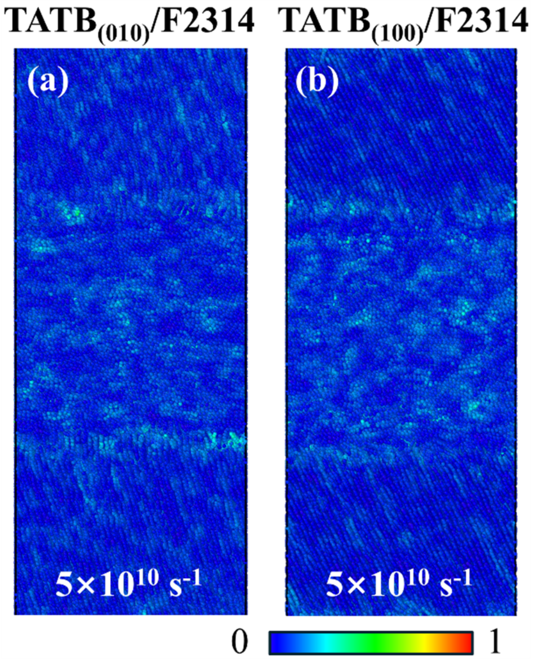

Figure S10. Peak-state shear strain fields for (a) $TATB_{(010)}$/F2314 and (b) $TATB_{(100)}$/F2314 at 5×10^{10} s^{-1} and 250 K.

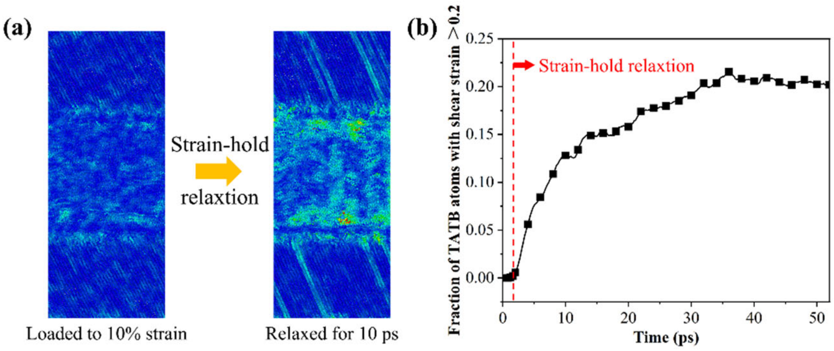

Figure S11. (a) Evolution of the TATB(100)/F2314 interfacial structure during strain-hold relaxation. The color scale represents the shear strain ranging from 0 to 1; (b) fraction of TATB atoms exhibiting a local von Mises shear strain greater than 0.20. The red dashed line indicates the end of the loading stage.

Layer-resolved characterization of basal-plane kinking

To clarify the molecular origin of the localized deformation band in TATB(001)/F2314, a layer-resolved displacement analysis was performed under the representative condition of 400 K and 1×10^{9} s^{-1}. The geometric center of each TATB molecule was used to characterize its translational motion. The non-affine displacement of molecule *i* was calculated as:

$$u_i^{NA}(t) = r_i(t) - r_{aff,i}(t) - u_s^{rigid}(t) \tag{S2}$$

where $r_i(t)$ is the current center of mass position of TATB molecule *i*, $r_{aff,i}(t)$ is its position obtained by applying the macroscopic affine deformation to the reference configuration, $u_s^{rigid}(t)$ is the median rigid translation of the corresponding TATB slab *s*. Periodic boundary conditions were considered when calculating molecular displacements. Within each layer, the non-affine displacements were locally averaged over a 3×3 in-plane neighborhood to reduce thermal fluctuations without averaging across adjacent molecular layers. The local relative displacement between layers *l* and *l*+1 was then calculated as:

$$\Delta u_l(x) = \overline{u}_{l+1}^{NA}(x) - \overline{u}_l^{NA}(x) \tag{S3}$$

where *x* denotes the local in-plane position. The basal and normal components of the relative displacement were obtained from:

$$\Delta u_l^{\parallel} = (I - n_{001} n_{001}^T)\Delta u_l \tag{S4}$$

and

$$\Delta u_l^{\perp} = \Delta u_l \cdot n_{001} \tag{S5}$$

where n_{001} is the normal to the (001) molecular layers.

The principal direction of the large basal interlayer-displacement vectors was determined from the second-moment tensor:

$$C = \sum_{j=1}^{N} \Delta u_j^{\parallel} \otimes \Delta u_j^{\parallel} \tag{S6}$$

The eigenvector corresponding to the largest eigenvalue of *C* defines the principal basal displacement direction. Its directional concentration was quantified using the principal displacement fraction:

$$f_{dir} = \frac{\lambda_{\max}}{tr(C)} \tag{S7}$$

where $\lambda_{\max}$ is the largest eigenvalue of *C*. The principal direction was compared with the [100], [210], [110], [120], and [010] directions in the (001) plane. The analysis was repeated using the upper 1-10% of the basal interlayer-displacement distribution to evaluate the sensitivity of the crystallographic assignment to the active-region threshold. The results are shown in Figure 11 and Table S3. The magnitude of localized interlayer deformation was characterized by the 95th percentile P_{95} of the distribution of local displacement jumps between adjacent layers. This upper-percentile measure captures the strongly deformed regions while reducing sensitivity to isolated extreme values.

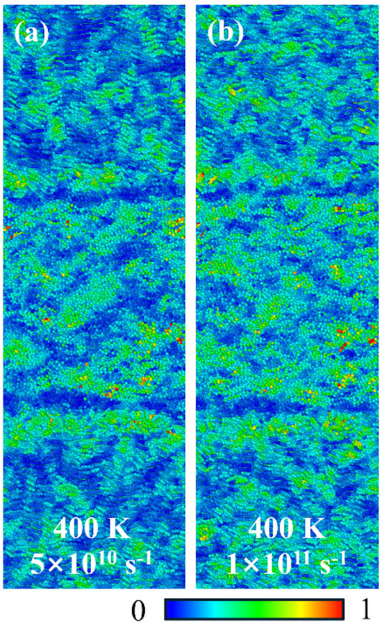

Figure S12. Peak-state shear strain fields of $TATB_{(001)}$/F2314 at 400 K under strain rates of (a) 5×10^{10} s^{-1} and (b) 1×10^{11} s^{-1}.

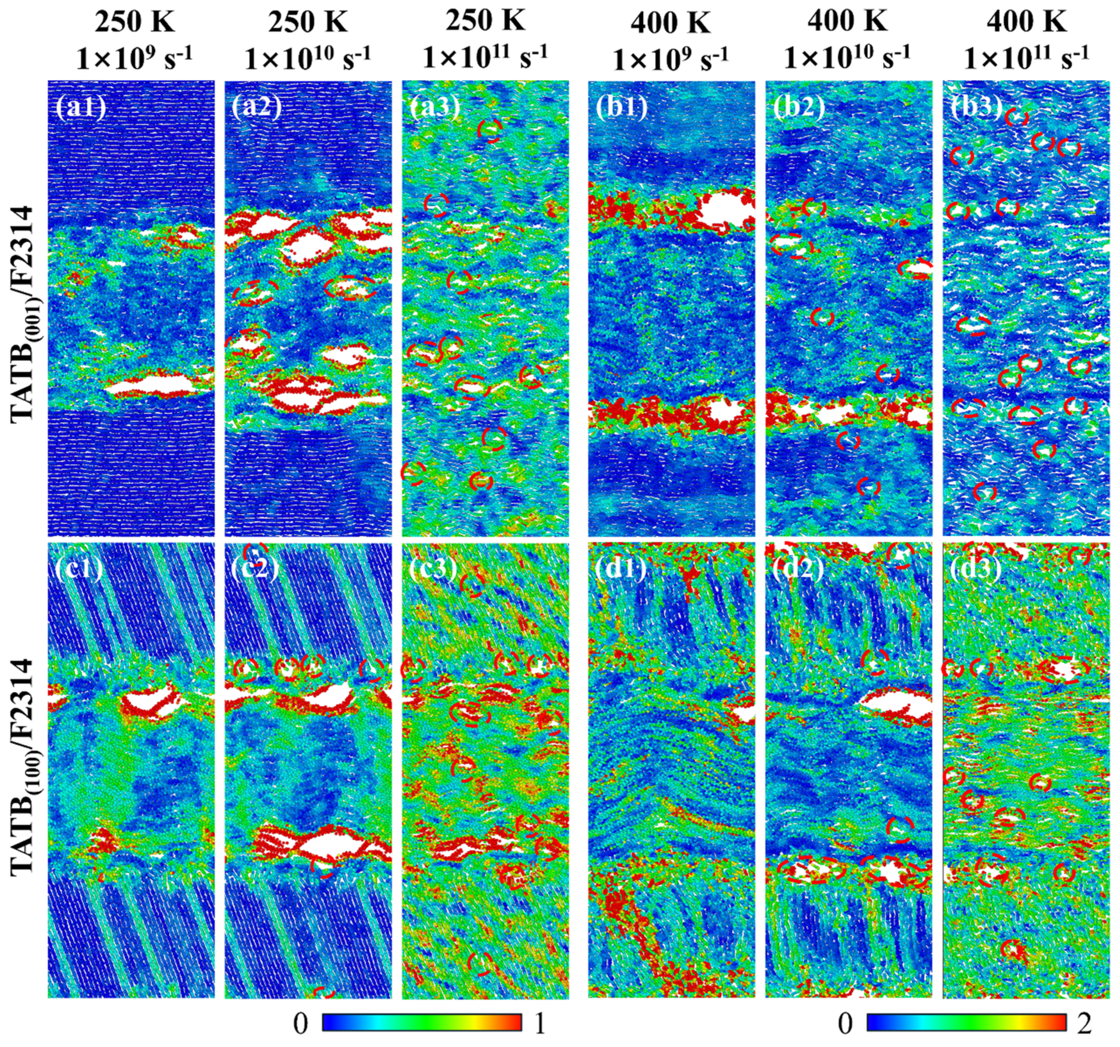

Figure S13. Post-peak damage morphologies of (a1-b3) $TATB_{(001)}$/F2314 and (c1-d3) $TATB_{(100)}$/F2314. All configurations correspond to the first frame on the descending branch closest to $\sigma / \sigma_{peak} \approx 0.5$. Atoms are colored according to the local von Mises shear strain and partial voids are highlighted by red circles. Different color scales are used to improve the visibility of local deformation: 0-1 for the 250 K panels and 0-2 for the 400 K panels.

Table S1. Potential Parameters of the TATB/fluoropolymer interface [16].

Morse	D_0 (eV)	α (Å^{-1})	r_0 (Å)	exp.	A (eV)	ρ (Å)
φ_{C-C}	0.0646	3.5455	2.7841	φ_{C-O}	4.4782	0.2624
φ_{C-H}	0.0082	1.3183	3.4755	φ_{C-N}	948.5813	0.3545
φ_{C-F}	0.0022	1.5916	3.9391	φ_{C-Cl}	813.4496	0.3331
φ_{H-H}	0.6905	2.7744	1.4724	φ_{H-Cl}	129.6145	0.3713
φ_{H-O}	0.0107	1.7749	2.9933	-	-	-
φ_{H-N}	0.000281	2.8725	2.0595	-	-	-
φ_{H-F}	0.0044	0.8713	3.6697	-	-	-
φ_{O-F}	0.000802	1.8822	3.8568	-	-	-
φ_{O-Cl}	0.0070	1.3180	3.3781	-	-	-
φ_{N-F}	0.0411	1.9042	2.7885	-	-	-
φ_{N-Cl}	0.3661	1.5199	2.2480	-	-	-

Table S2. Directional projection indices along the [120] and [210] directions.

Model	Strain rate	$\eta_{[120]}$	$\eta_{[210]}$
$TATB_{(010)}$/F2314	$1\times10^{10}\ s^{-1}$	0.977	0.305
$TATB_{(100)}$/F2314	$1\times10^{10}\ s^{-1}$	0.169	0.702

*For $TATB_{(010)}$/F2314, the directional projection index along [120] reaches 0.977, substantially exceeding the corresponding value along [210]. In contrast, $TATB_{(100)}$/F2314 exhibits larger projection indices along [210] than along [120]. Together with the localization of the high shear strain bands between adjacent molecular layers, these results support the assignment of the active slip systems as (001)[120] for $TATB_{(010)}$/F2314 and (001)[210] for $TATB_{(100)}$/F2314.

Table S3. Layer-resolved displacement characteristics of the kink band in $TATB_{(001)}$/F2314 at 400 K and 1×10^9 s^{-1}.

TATB slab	Basal P_{95} (Å)	Normal P_{95} (Å)	Angle to [100]	f_{dir}
1	6.27	1.30	4.18°	0.984
2	8.54	1.42	0.49°	0.989
